# The Prevalence of Nutritional Disorders in Inflammatory Bowel Disease: A Systematic Review and Meta-Analysis

**DOI:** 10.1016/j.advnut.2026.100758

**Published:** 2026-10-17

**Authors:** Tong Chen, Zhou Zhou, Mingyue Guo, Yan Pan, Zhenzhen Guo, Wenhui Xie, Shiyu Xiao

**Affiliations:** 1 Department of Rheumatology and Clinical Immunology, Peking University First Hospital, Beijing, China; 2 School of Medicine, University of Electronic Science and Technology of China, Chengdu, China; 3 Department of Gastroenterology, Sichuan Provincial People’s Hospital, School of Medicine, University of Electronic Science and Technology of China, Chengdu, China; 4 Department of Gastroenterology, Clinical Immunology Translation Medicine Key Laboratory of Sichuan Province, Sichuan Provincial People’s Hospital, University of Electronic Science and Technology of China, Chengdu, China

**Keywords:** underweight, malnutrition, overweight, obesity, low muscle mass, sarcopenia, inflammatory bowel disease, prevalence, meta-analysis

## Abstract

The epidemiology of nutritional disorders in inflammatory bowel disease (IBD) remains poorly characterized. Existing evidence is fragmented across specific conditions or confined to single regions, and no study has simultaneously assessed the principal nutritional disorders—malnutrition, overweight or obesity, and sarcopenia—in a unified framework. To address this gap, we systematically searched PubMed, Embase, Cochrane Library, and China Biology Medicine from inception to 31 July, 2024 and updated on 30 July, 2026. Observational studies reporting prevalence based on standard diagnostic criteria were included. Pooled estimates with 95% confidence interval (CI) and prediction interval (PI) were derived using a random-effects model. Prespecified subgroup analyses were conducted by sex, age, disease subtype and activity, diagnostic criteria, study setting, and geographic variation. Study quality and publication bias were also assessed. After literature screening, 201 studies across 32 countries were included. Underweight affected 11.5% (95% CI: 9.0%, 14.6%, 95% PI: 1.0%, 61.7%), whereas consensus-based malnutrition (Global Leadership Initiative on Malnutrition, European Society for Clinical Nutrition and Metabolism, Mini Nutritional Assessment, Subjective Global Assessment or its variant Patient-Generated -Subjective Global Assessment) affected 27.6% (95% CI: 20.5%, 36.1%, 95% PI: 3.6%, 79.7%). Notably, this malnutrition estimate was derived almost exclusively from hospital-based settings (>80%) and predominantly from a single region. Overweight and obesity affected 23.1% (95% CI: 20.6%, 25.9%, 95% PI: 8.1%, 50.8%) and 13.5% (95% CI: 11.7%, 15.6%, 95% PI: 2.9%, 44.6%), respectively. For muscle depletion, low muscle mass defined by skeletal muscle index was identified in 48.3% (95% CI: 41.8%, 55.0%, 95% PI: 19.7%, 78.1%), whereas consensus-based sarcopenia (European Working Group on Sarcopenia in Older People or Asian Working Group on Sarcopenia) affected 12.6% (95% CI: 6.1%, 24.2%, 95% PI: 0.9%, 70.6%). Of note, the majority of included studies for malnutrition and sarcopenia were hospital based. Active disease was associated with a significantly higher prevalence of malnutrition. No significant differences were observed between disease subtypes. In conclusion, our findings provide a broad overview of the prevalence of major nutritional disorders in patients with IBD, highlighting that nutritional care in IBD should extend beyond simple weight monitoring. However, given the wide PIs, these pooled estimates reflect the overall level across highly heterogeneous settings rather than a precise global figure. Future research, particularly population-based studies with standardized diagnostic criteria across diverse regions, is needed to validate our observation.

The study protocol was registered a priori at PROSPERO as CRD42024592104.

**Statement of significance**

To our knowledge, this first comprehensive meta-analysis quantifies the prevalence of nutritional disorders (underweight, malnutrition, overweight or obesity, low muscle mass, and sarcopenia) in the population with inflammatory bowel disease (IBD), revealing substantial variation by diagnostic criteria and clinical factors. The findings underscore the need for standardized, multidimensional nutritional assessment in IBD management.

## Introduction

Inflammatory bowel diseases (IBD), comprising Crohn’s disease (CD) and ulcerative colitis (UC), are chronic, disabling conditions of the gastrointestinal tract whose global prevalence is rising steadily, establishing them as a worldwide public health challenge [1], [2]. The therapeutic landscape for IBD has been revolutionized by biologic and small-molecule agents, shifting treatment paradigms toward more ambitious endpoints such as mucosal healing [3], [4]. Yet, amidst these advances, the fundamental domain of nutrition—a critical determinant of immune competence, tissue repair, and overall physiological resilience—remains a pivotal yet often undermanaged aspect of comprehensive care in patients with IBD [5].

Nutritional health in IBD spans a complex spectrum, from micronutrient deficiencies to profound body composition changes. Central to this pathophysiology are 3 principal, often interlinked disorders: malnutrition, driven by inflammation and malabsorption; overweight or obesity, reflecting the confluence of chronic disease and the global obesity epidemic; and sarcopenia, the loss of muscle mass and function that undermines physical resilience [5]. The clinical relevance of these disorders is profound. Malnutrition impairs immune function and tissue repair, directly contributing to increased morbidity [6]. Obesity, particularly visceral obesity, functions as a proinflammatory endocrine organ, potentially exacerbating disease activity and diminishing response to biologic therapies [7]. Sarcopenia is a powerful predictor of postoperative complications, therapeutic failure, and reduced quality of life [8], [9]. Given these powerful associations with adverse outcomes, international guidelines now mandate regular nutritional screening in IBD [10].

Despite its clinical imperative, the epidemiology of these foundational nutritional disorders remains profoundly fragmented. Existing literature presents a highly variable prevalence estimates—for instance, malnutrition is reported anywhere from 6.1% to 92.5% [5]—obscured by heterogeneous study populations, divergent diagnostic criteria, and a narrow geographic focus. Recent systematic reviews have attempted to synthesize evidence on individual nutritional disorders. For example, 1 review reported that >40% of patients with IBD exhibit low muscle mass, with nearly one-fifth meeting sarcopenia criteria [8]. However, no study to date has systematically quantified the prevalence of the core nutritional disorders—malnutrition, obesity, and muscle impairment—within a unified framework, nor has it examined how their estimates vary across different diagnostic criteria and geographic settings. This evidence gap leaves the relative burden, geographic distribution, and clinical determinants of these disorders largely undefined, thereby constraining the development of evidence-based, region-adapted nutritional care strategies.

To address these critical knowledge gaps, we conducted a systematic review and meta-analysis to determine the prevalence of the 3 principal nutritional disorders—malnutrition, overweight or obesity, and muscle depletion—in patients with IBD. Furthermore, we sought to dissect the epidemiological heterogeneity by examining key demographic and clinical determinants. By providing this comprehensive estimate, our study aimed to establish a foundational resource for understanding the etiology, consequences, and management of nutritional disorders in IBD worldwide.

## Methods

### Study registration

This systematic review and meta-analysis were conducted in accordance with the PRISMA guidelines [11]. The study protocol was registered a priori with PROSPERO (CRD42024592104).

### Search strategy and data sources

A systematic literature search of 4 electronic databases—PubMed, Embase, Cochrane Library, and China Biology Medicine database—was performed from their inception to 31 August, 2024 and updated in 30 July, 2026 without restrictions on language. The search strategy was designed around core concepts of the population (“inflammatory bowel disease,” “Crohn’s disease,” and “ulcerative colitis”) and the outcomes of interest (“malnutrition,” “undernutrition,” “sarcopenia,” “overweight,” and “obesity”). Supplemental Appendix 1 provides the full details of the retrieval strategy. To ensure comprehensiveness, the reference lists of included studies and relevant review articles were manually screened.

### Eligibility criteria and definitions

Studies were included if they met the following criteria: *1*) observational longitudinal design (cohort or cross-sectional); *2*) enrolled a confirmed IBD population; *3*) reported the prevalence of, or provided raw data to calculate, at least one of the predefined nutritional disorders: underweight, malnutrition, overweight or obesity, low muscle mass, and sarcopenia; and *4*) specified the diagnostic criteria used for the nutritional assessment. Studies were excluded if they had a sample size of <100 patients (to minimize the likelihood of overestimating the magnitude of the issue because of a small sample size) [12]. Reviews, conference abstracts, case reports, or commentaries were also not included. The specific definitions and available diagnostic criteria used to categorize each nutritional disorder are detailed in Supplemental Appendix 2.

### Data extraction, outcome, and quality assessment

Two independent reviewers (Tong Chen and Zhou Zhou) extracted data using a prepiloted, standardized form. Any discrepancies were resolved through consensus or by a third reviewer (Mingyue Guo). Extracted data included the following: first author, publication year, country, WHO region, sample size, age, sex, disease subtype (CD compared with UC), disease activity (active compared with remission), diagnostic criteria for nutritional disorders, and the raw counts for calculating prevalence.

The primary outcomes were the pooled prevalence of underweight, malnutrition, overweight or obesity, and muscle impairment (low muscle mass and sarcopenia). Secondary outcomes included the prevalence of these nutritional disorders grouped by sex, age group (children or adolescents compared with adults), disease subtype (CD compared with UC), disease activity (active compared with remission), diagnostic criteria [for consensus-defined malnutrition: Global Leadership Initiative on Malnutrition (GLIM), European Society for Clinical Nutrition and Metabolism (ESPEN), Mini Nutritional Assessment (MNA), Subjective Global Assessment (SGA) or its variant Patient-Generated SGA (PG-SGA); for consensus-based sarcopenia: European Working Group on Sarcopenia in Older People (EWGSOP), the Asian Working Group for Sarcopenia (AWGS)], and study setting (hospital based compared with population based). The exploratory analysis of geographical patterns of these nutrional disorders was also performed.

The methodological quality of cross-sectional studies was assessed using the 11-item tool from the Agency for Healthcare Research and Quality [13]. For longitudinal studies, the Newcastle-Ottawa Scale was used [14].

### Data synthesis and analysis

All statistical analyses were performed using R software (version 4.3.2) [15]. Prevalence estimates with 95% confidence interval (95% CI) were pooled using a random-effects model via the generalized linear mixed model (GLMM) framework to generate more robust CIs for proportional data [16]. Prediction intervals (PIs) were also calculated for all primary pooled estimates to provide a measure of the expected range of true effects in future individual studies. Heterogeneity was quantified using the *I*^2^ statistic, with an *I*^2^ value >50% considered to represent substantial heterogeneity. Prespecified subgroup analyses were conducted to explore sources of heterogeneity. For subgroup comparisons, odds ratios (ORs) with 95% CIs were calculated by dividing the pooled prevalence estimate of 1 subgroup (e.g., male) by that of the reference subgroup (e.g., female), using the log-transformed proportions and their SEs derived from the GLMM for each subgroup. Leave-one-out sensitivity analysis was performed to evaluate whether any single study disproportionately influenced the pooled estimates. Publication bias was assessed using funnel plots and Egger’s linear regression test for outcomes with ≥10 included studies [17]. A 2-tailed *P* value < 0.05 was considered statistically significant for all analyses.

## Results

### Study selection and characteristics

In our initial and updated search, we identified 18,882 records from the designated databases. After duplicate removal, 12,220 records underwent title and abstract screening. After full-text assessment of 740 articles, 201 studies finally met eligibility criteria and were included in the quantitative synthesis (Figure 1). Study characteristics are summarized in Supplemental Table 1. Methodological quality was generally moderate to high (Supplemental Table 1).

**FIGURE 1:**
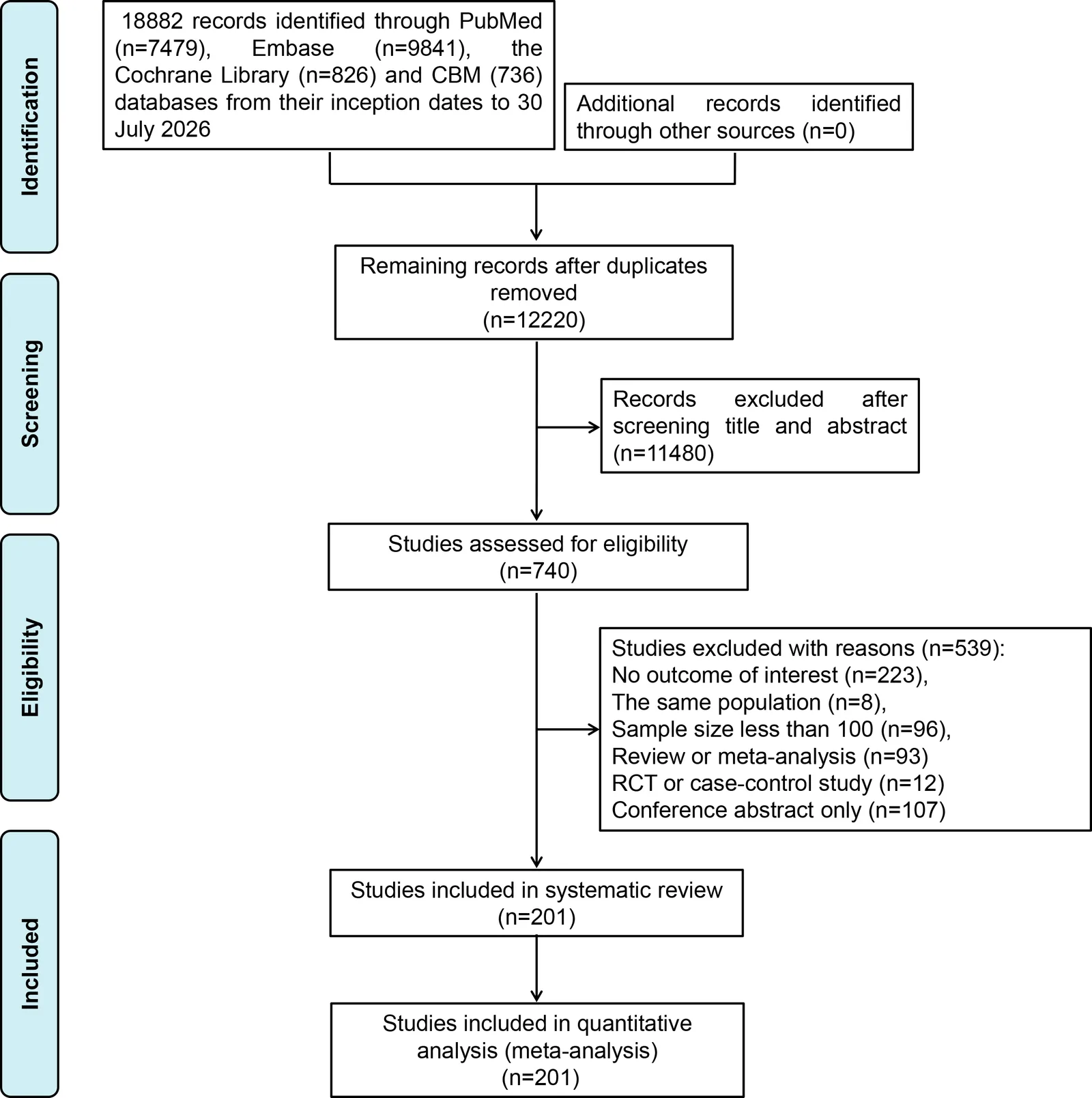
Flow diagram of the study selection process. CBM, China Biology Medcine; RCT, Randomized Clinical Trial.

### Prevalence of nutritional disorders in patients with IBD

#### Underweight

The pooled prevalence of underweight in patients with IBD was 11.5% (95% CI: 9.0%, 14.6%; 95% PI: 1.0%, 61.7%; 89 studies, *I*^2^ = 99%) (Table 1). Leave-one-out analysis confirmed the robustness of this estimate (postexclusion range: 11.1%–11.9%). Visual inspection of the funnel plot suggested asymmetry (Supplemental Figure 1A), which was confirmed by Egger’s test (*P* = 0.031). Further trim-and-fill adjustment yielded a similar estimate of 9.7% (95% CI: 7.4%, 12.5%) (Supplemental Figure 1B), indicating the stability of the result.

**TABLE 1:** Prevalence of nutritional disorders in patients with IBD

	Studies (*n*)	Sample size	Prevalence (95% CI)	95% PI	*I*^2^ (%)
Underweight	89	128,898	11.5 (9.0, 14.6)	1.0, 61.7	99
Consensus-defined malnutrition	35	34,193	27.6 (20.5, 36.1)	3.6, 79.7	99
Overweight	69	150,202	23.1 (20.6, 25.9)	8.1, 50.8	98
Obesity	100	1,393,054	13.5 (11.7, 15.6)	2.9, 44.6	100
Visceral obesity (imaging defined)	4	518	28.6 (20.7, 38.2)	14.3, 49.2	83
Low muscle mass (defined by SMI)	26	5393	48.3 (41.8, 55.0)	19.7, 78.1	94
Low muscle mass with obesity	3	1269	5.7 (4.5, 7.1)	4.5, 7.1	43
Consensus-defined sarcopenia	12	6874	12.6 (6.1, 24.2)	0.9, 70.6	98
Sarcopenic obesity	1	110	12.7 (7.1, 20.4)	—	—

Abbreviations: CI, confidence interval; IBD, inflammatory bowel disease; PI, prediction interval; SMI, skeletal muscle index.

#### Consensus-based malnutrition

For malnutrition defined by consensus criteria (GLIM, ESPEN, MNA, or SGA/PG-SGA), the pooled prevalence in patients with IBD was 27.6% (95% CI: 20.5%, 36.1%; 99% PI: 3.6%, 79.7%; 35 studies, *I*^2^ = 99%) (Table 1). Sensitivity analysis pooled consistent estimates (postexclusion range: 26.6%–29.6%). Visual inspection of the funnel plot (Supplemental Figure 2A) and Egger's test (*P* = 0.11) did not suggest publication bias.

#### Overweight, obesity, and visceral obesity

##### Overweight and obesity

The combined prevalence of overweight or obesity was 28.0 (95% CI: 24.4, 31.9; 95% PI: 4.8, 75.0; 122 studies, *I*^2^ = 100%). Specifically, the pooled prevalence of overweight in IBD was 23.1% (95% CI: 20.6%, 26.0%; 95% PI: 8.1%, 50.8%; 69 studies, *I*^2^ = 98%) (Table 1), whereas obesity had a prevalence of 13.5% (95% CI: 11.7%, 15.6%; 95% PI: 2.9%, 44.6%; 100 studies, *I*^2^ = 100%) (Table 1). Leave-one-out sensitivity analysis confirmed the stability of both pooled estimates. Funnel plot and Egger’s test showed no evidence of publication bias for obesity alone (*P* = 0.051), whereas asymmetry was suggested for overweight (Egger’s test: *P* = 0.032) (Supplemental Figure 3). Trim-and-fill adjustment for overweight produced a similar estimate at 28.7% (95% CI: 25.0%, 32.7%).

##### Visceral obesity

Visceral obesity assessed by imaging yielded a prevalence of 28.6% (95% CI: 20.7%, 38.2%; 4 studies, *I*^2^ = 83%) (Table 1, Supplemental Figure 4).

#### Muscle depletion

##### Low muscle mass

The pooled prevalence of low muscle mass defined by skeletal muscle index was 48.3% (95% CI: 41.8%, 55.0%; 95% PI: 19.7%, 78.1%; 26 studies, *I*^2^ = 94%) (Table 1). Sensitivity analysis produced a consistent estimate (postexclusion range: 47.1%–49.8%). No publication bias was found by the funnel plot (Supplemental Figure 5) and Egger's test (*P* = 0.48). Besides, the pooled prevalence of low muscle mass with obesity was 5.7% (95% CI: 4.5%, 7.1%; 95% PI: 4.5%, 7.1%; 3 studies, *I*^2^ = 58%) (Supplemental Figure 6).

##### Sarcopenia

For sarcopenia diagnosed by consensus criteria (EWGSOP or AWGS), the pooled prevalence was 12.6% (95% CI: 6.1%, 24.2%; 95% PI: 0.9%, 70.6%; 12 studies, *I*^2^ = 98%) (Table 1). Leave-one-out analysis pooled consistent results (postexclusion range: 13.4%–19.0%). No publication bias was found by the funnel plot (Supplemental Figure 6) and Egger's test (*P* = 0.41).

##### Sarcopenic obesity

One study reported the prevalence of sarcopenic obesity was 12.7% (95% CI: 7.1%, 20.4%) [18].

#### Prevalence of nutritional disorders based on population demographics and disease characteristics

##### Sex (male compared with female)

No significant sex differences were observed for underweight, malnutrition, obesity, low muscle mass, or sarcopenia (all *P* > 0.05), whereas overweight was more common in males than females [30.4% compared with 23.8%; OR = 1.41 (95% CI: 1.03, 1.93), *P* = 0.03] (TABLE 2, TABLE 3, TABLE 4, TABLE 5).

**TABLE 2:** Prevalence of underweight grouped by population demographics and disease characteristics

	Studies (*n*)	Sample size	Prevalence (95% CI)	*I*^2^ (%)	*P* for subgroup	OR	*P*
Sex	—	—	—	—	0.14	—	
Female	19	9440	8.5 (4.8, 14.6)	96		Ref	
Male	19	9253	4.8 (2.8, 8.1)	94		0.53 (0.23, 1.22)	0.14
Population group	—	—	—	—	0.07	—	
Children or adolescent	16	7177	18.9 (12.4, 27.7)	97		Ref	
Adult	60	108,896	10.4 (7.7, 13.8)	99		0.50 (0.26, 0.97)	0.04
Mixed	8	1728	12.1 (6.2, 22.2)	94		0.58 (0.21, 1.64)	0.31
Disease subtype	—	—	—	—	0.03	—	
UC	34	10,457	8.8 (6.1, 12.5)	95		Ref	
CD	51	29,830	14.6 (10.8, 19.4)	99		1.78 (1.05, 3.04)	0.03
Disease activity	—	—	—	—	0.06	—	
Remission	4	742	5.0 (2.9, 8.6)	66		Ref	
Active	6	1186	12.4 (5.8, 24.7)	93		3.03 (0.96, 9.59)	0.06

Abbreviations: CD, Crohn’s disease; CI, confidence interval; IBD, inflammatory bowel disease; OR, odds ratio; Ref., reference; UC, ulcerative colitis.

**TABLE 3:** Prevalence of consensus-defined malnutrition grouped by population demographics and disease characteristics

	Studies (*n*)	Sample size	Prevalence (95% CI)	*I*^2^	*P* for subgroup	OR	*P*
Sex	—	—	—	—	0.45	—	
Female	14	14,087	33.3 (19.8, 50.2)	99%		Ref	
Male	14	15,139	25.8 (15.6, 39.5)	99%		0.69 (0.27, 1.79)	0.45
Population group	—	—	—	0.68		—	
Children or adolescent	—	—	—	—		—	
Adult	33	33,700	28.0 (20.7, 36.8)	99%		—	
Mixed	2	493	21.3 (4.8, 59.3)	99%		—	
Disease subtype	—	—	—	—	0.40	—	
UC	19	14,128	19.6 (12.3, 30.0)	99%		Ref	
CD	19	17,633	25.6 (16.4, 37.7)	99%		1.41 (0.64, 3.11)	0.40
Disease activity	—	—	—	—	<0.01	—	
Remission	8	1310	18.8 (9.4, 34.1)	96%		Ref	
Active	12	2305	47.8 (34.5, 61.4)	97%		3.93 (1.53, 10.11)	<0.01

Abbreviations: CD, Crohn’s disease; CI, confidence interval; IBD, inflammatory bowel disease; OR, odds ratio; Ref., reference; UC, ulcerative colitis.

**TABLE 4:** Prevalence of overweight and obesity grouped by population demographics and disease characteristics

	Overweight							Obesity						
	Studies (*n*)	Sample size	Prevalence (95% CI)	*I*^2^ (%)	*P* for subgroup	OR	*P*	Studies (*n*)	Sample size	Prevalence (95% CI)	*I*^2^ (%)	*P* for subgroup	OR	*P*
Sex	—	—	—	—	0.04	—		—	—	—	—	0.31	—	
Female	21	15,934	23.8 (20.3, 27.8)	94		Ref		35	466,208	15.4 (12.1, 19.4)	100		Ref	
Male	21	16,340	30.4 (25.7, 35.6)	95		1.41 (1.03, 1.93)	0.03	35	379,065	12.8 (9.9, 16.5)	100		0.81 (0.54, 1.22)	0.32
Population group	—	—	—	—	<0.01	—		—	—	—	—	<0.01	—	
Children or adolescent	10	10,199	10.3 (6.6, 15.6)	95		Ref		11	22915	4.9 (3.2, 7.5)	98		Ref	
Adult	50	126,975	25.6 (23.3, 28.1)	98		2.97 (2.10, 4.19)	<0.01	76	1,040,322	14.8 (12.9, 17.1)	100		3.40 (2.10, 5.49)	<0.01
Mixed	5	1667	26.3 (23.9, 29.0)	46		3.04 (1.78, 5.21)	<0.01	9	318,456	14.7 (9.2, 22.6)	100		3.37 (1.74, 6.2)	<0.01
Disease subtype	—	—	—	—	0.11	—		—	—	—	—	0.17	—	
UC	29	21,762	24.5 (20.3, 29.2)	96		Ref		43	342,249	15.0 (12.3, 18.1)	100		Ref	
CD	39	43,140	19.8 (16.3, 23.8)	97		0.76 (0.54, 1.08)	0.12	53	406,488	12.3 (10.1, 15.0)	99		0.80 (0.58, 1.11)	0.18
Disease activity	—	—	—	—	0.49	—		—	—	—	—	0.88	—	
Remission	5	2520	21.1 (13.4, 31.63)	97		Ref		5	2520	9.7 (5.2, 17.4)	94		Ref	
Active	6	3674	17.1 (10.8, 25.9)	93		0.77 (0.36, 1.64)	0.49	6	3674	10.3 (5.6, 18.4)	94		1.07 (0.41, 2.80)	0.88

Abbreviations: CD, Crohn’s disease; CI, confidence interval; IBD, inflammatory bowel disease; OR, odds ratio; Ref., reference; UC, ulcerative colitis.

**TABLE 5:** Prevalence of muscle depletion grouped by population demographics and disease characteristics

	Low muscle mass (SMI-defined)							Sarcopenia (EWGSOP/AWGS)						
	Studies (*n*)	Sample size	Prevalence (95% CI)	*I*^2^ (%)	*P* for subgroup	OR	*P*	Studies (*n*)	Sample size	Prevalence (95% CI)	*I*^2^ (%)	*P* for subgroup	OR	*P*
Sex	—	—	—	—	0.07	—		—	—	—	—	0.61	—	
Female	18	1215	42.8 (34.7, 51.3)	85		Ref		3	136	35.4 (20.1, 54.3)	83		Ref	
Male	18	2028	54.9 (44.6, 64.9)	93		1.63 (0.95, 2.80)	0.08	3	203	30.0 (20.2, 41.9)	77		0.77 (0.31, 1.92)	0.57
Disease subtype	—	—	—	—	0.42	—		—	—	—	—	0.31	—	
UC	5	990	48.9 (45.8, 52.0)	57		Ref		7	4158	7.0 (2.9, 15.8)	97		Ref	
CD	18	3429	52.4 (44.4, 60.3)	94		1.36 (0.72, 2.58)	0.34	9	2863	14.7 (4.4, 39.6)	97		2.31 (0.42, 12.6)	0.33
Disease activity	—	—	—	—	0.08	—		—	—	—	—	0.12	—	
Remission	4	214	42.6 (28.1, 58.5)	83		Ref		6	905	11.6 (5.0, 24.7)	92		Ref	
Active	5	994	60.0 (49.0, 70.1)	86		2.03 (0.95, 4.31)	0.07	5	362	29.2 (11.7, 56.2)	88		3.21 (0.76, 13.53)	0.11

Abbreviations: AWGS, Asian Working Group on Sarcopenia; CD, Crohn’s disease; CI, confidence interval; EWGSOP, European Working Group on Sarcopenia in Older People; IBD, inflammatory bowel disease; OR, odds ratio; Ref., reference; SMI, skeletal muscle index; UC, ulcerative colitis.

##### Age groups

Underweight showed no significant age-related differences (*P* = 0.07), although children/adolescents had a higher point estimate [18.9% compared with 10.4% in adults; OR = 0.58 (95% CI: 0.21, 1.64), *P* = 0.31] (Table 2). Overweight or obesity was predominantly an adult issue, with prevalence significantly higher in adults than in children/adolescents [overweight: 25.6% compared with 10.3%; OR = 2.97 (95% CI: 2.10, 4.19), *P* < 0.01; obesity: 14.8% compared with 4.9%; OR = 3.37 (95% CI: 1.74, 6.52), *P* < 0.01] (Table 4). Data on malnutrition and sarcopenia across age groups were insufficient for further analysis.

##### Disease subtype

Underweight appeared more common in CD than UC (14.6% compared with 8.8%, *P* for subgroup = 0.03; OR = 1.78 (95% CI: 1.05, 3.04), *P* = 0.03], but no significant differences between disease subtypes were observed for most outcomes (TABLE 2, TABLE 3, TABLE 4, TABLE 5).

##### Disease activity

Disease activity was a key determinant of nutritional status. Malnutrition was significantly more prevalent in active disease than remission [47.8% compared with 18.8%, *P* for subgroup < 0.01; OR = 3.93 (95% CI: 1.53, 10.11), *P* < 0.01] (Table 3). The prevalence of underweight (15.8% compared with 5.0%, *P* for subgroup = 0.02; OR = 4.11 (95% CI: 1.13, 15.02), *P* = 0.03) (Table 2), low muscle mass [60.0% compared with 42.6%, *P* for subgroup = 0.06; OR: 2.03 (95% CI: 0.95, 4.31), *P* = 0.07], and sarcopenia [29.2% compared with 11.6%, *P* for subgroup < 0.01; OR =3.21 (95% CI: 0.76, 13.53), *P* = 0.11] also trended higher in active disease, although the difference did not reach statistical significance (TABLE 2, TABLE 5 and 5). In contrast, overweight or obesity prevalence was similar regardless of disease activity (overweight: 17.1% compared with 21.1%, *P* for subgroup = 0.49; obesity: 10.4% compared with 9.7%, *P* for subgroup = 0.88) (Table 4).

### Prevalence of nutritional disorders grouped by study characteristics

#### Diagnostic criteria

Given the heterogeneity in definitions of malnutrition and sarcopenia of included studies, we further pooled the prevalence estimates according to the specific diagnostic criteria applied. For malnutrition, GLIM-based criteria yielded the highest estimate at 36.9% (95% CI: 27.1%, 47.9%), followed by SGA/PG-SGA at 25.1% (95% CI: 13.9%, 41.2%), ESPEN at 24.0% (95% CI: 13.0%, 40.2%), and MNA at 13.3% (95% CI: 8.3%, 20.8%) (*P* for subgroup <0.01) (Supplemental Figure 7). Despite these differences, the subgroup patterns regarding population demographics (sex and age) and disease characteristics (disease subtype and activity) remained consistent with the overall pooled results (Supplemental Table 2). Besides, for malnutrition diagnosed via International Classification of Diseases coding, the pooled prevalence was 11.4% (95% CI: 8.2%, 15.8%; 4 studies, *I*^2^ = 100%), based on a total population of ∼2.3 million (Supplemental Figure 8). For sarcopenia, EWGSOP criteria produced a prevalence of 6.2% (95% CI: 2.3%, 15.5%), whereas AWGS criteria yielded a substantially higher estimate of 29.7% (95% CI: 23.2%, 37.0%) (Supplemental Figure 9). Nevertheless, both criteria consistently indicated a similar pattern in terms of sex, disease subtype, or disease activity (Supplemental Table 3).

#### Study setting

Given that >80% of included studies were hospital based, we also conducted a subgroup analysis by study settings. The prevalence estimates for underweight (6.2%), overweight (25.0%), and obesity (14.0%) in population-based studies were broadly consistent with the main analyses, with similar patterns across sex, age, and disease subtype (Supplemental Tables 4 and 5). For instance, overweight and obesity were more prevalent in adults than in children or adolescents (*P* for group < 0.01) (Supplemental Table 5). Notably, population-based studies reporting malnutrition or sarcopenia were scarce, precluding robust subgroup analyses for these outcomes.

### Exploratory analysis of geographic pattern of nutritional disorders in patients with IBD

Our included studies spanned 32 countries across 6 WHO regions, with underrepresentation from Africa and South America. Thus, as an exploratory analysis, we also assessed the geographic patterns of these nutritional disorders at the regional and country levels. The Western Pacific Region bore the highest burden of underweight (27.0%, 95% CI: 20.1%, 35.2%) (Supplemental Table 6), malnutrition (40.0%, 95% CI: 29.5%, 50.8%) (Supplemental Table 7), low muscle mass (53.9%, 95% CI: 47.0%, 60.7%) (Supplemental Table 8), and sarcopenia (28.9%, 95% CI: 20.9%, 38.5%) (Supplemental Table 9) with significantly higher odds compared with the Region of the Americas or European Region (Supplemental Table 10). Conversely, overweight and obesity were most prevalent in the Region of the Americas (26.2% and 16.5%, respectively) and the European Region (24.0% and 11.0%), whereas the Western Pacific Region had the lowest estimates (14.9% and 7.3%) (Supplemental Tables 11 and 12). The Eastern Mediterranean Region also showed a high prevalence of underweight (19.9%, 95% CI: 6.0%, 49.4%), although data were limited to 5 studies (Supplemental Table 2). No studies were identified from the African Region for any outcome. This regional pattern was mirrored at the country level, where notable disparities in prevalence estimates emerged across individual nations. The highest prevalence of underweight or malnutrition was predominantly observed in developing economies of Asia, particularly China (underweight: 35.4%; malnutrition: 43.7%), as well as in select countries in the Middle East (Saudi Arabia: 40.6% for underweight) (Supplemental Tables 6 and 7). Regarding overweight or obesity, Greece (34.6%) and the United Kingdom (31.6%) reported the highest overweight prevalence, whereas obesity was most prevalent in Australia (25.1%) and Greece (27.0%) (Supplemental Tables 11 and 12). In terms of muscle impairment at the national level, the highest prevalence was observed in South Korea (62.5%), Germany (59.7%), and China (55.1%) for low muscle mass, and China (33.0%) for sarcopenia (Supplemental Tables 8 and 9).

## Discussion

This comprehensive meta-analysis provides the pooled prevalence of the nutritional disorders, including underweight, malnutrition, overweight or obesity, low muscle mass, and sarcopenia, in patients with IBD. The patterns of these nutritional disorders are shaped by a confluence of clinical and diagnostic factors. However, the wide PIs across most outcomes indicate that these pooled figures represent the overall level across highly heterogeneous settings rather than a precise global prevalence estimate.

Malnutrition in IBD arises from inflammation-driven catabolism, malabsorption, and reduced dietary intake processes that can operate across the entire weight spectrum [5]. The marked discrepancy between prevalence estimates based on underweight (11.5%) and consensus-based malnutrition criteria (27.6%) underscores that BMI alone substantially underestimates the true burden of malnutrition in IBD, consistent with recent observations that BMI-based screening frequently misses malnutrition in patients with preserved body weight [19], [20]. Thus, BMI alone is an inadequate screening tool, reinforcing the need for comprehensive nutritional assessment in routine IBD care. The global ascendancy of overweight and obesity, now affecting nearly a quarter of the IBD population, signals a critical epidemiologic shift. This mirrors the general population’s obesity pandemic but carries unique implications for IBD [7]. It has been indicated that beyond serving as an energy reservoir, adipose tissue, particularly visceral fat, functions as a dynamic endocrine organ capable of secreting a range of proinflammatory cytokines and adipokines [21].

Sarcopenia in IBD is likely influenced by a synergy of chronic inflammation, nutrient malabsorption, and reduced physical activity [9]. The high prevalence of muscle impairment, establishing myopenia as a central feature of this disease, although the estimate varied dramatically depending on definition: nearly half of patients exhibited low muscle mass, whereas only 1 in 8 met consensus-based criteria for sarcopenia (EWGSOP/AWGS). This marked discrepancy highlights that the true burden of clinically significant sarcopenia, defined by both low muscle mass and functional impairment, may be substantially lower than previously reported based on muscle mass alone. Regarding the overlap of muscle depletion and obesity, limited data (3 studies on low muscle mass with obesity and 1 on sarcopenic obesity) preclude robust estimation.

The subgroup analyses further provide crucial clinical context for these epidemiological patterns. The powerful link between active diseases and malnutrition and low muscle mass was significantly more prevalent in active disease than in remission, which might reaffirm systemic inflammation as a driver of tissue wasting. In contrast, the stability of overweight and obesity across disease activity states suggests its development might be independent of acute flares. The lack of a significant sex disparity across most disorders may imply that the systemic inflammatory burden of IBD may override typical sex-specific differences in body composition. Notably, the choice of diagnostic criteria led to widely divergent absolute prevalence estimates. For malnutrition, GLIM-based criteria yielded the highest estimate (36.9%), followed by SGA/PG-SGA (25.1%), ESPEN (24.0%), and MNA (13.3%). This hierarchy likely reflects the increasing comprehensiveness and sensitivity of each tool. GLIM incorporates both phenotypic and etiologic criteria, making it more sensitive to disease-related catabolism [22]; whereas ESPEN and MNA rely more heavily on BMI and dietary intake, which may miss malnutrition in patients with preserved weight [23], [24]. SGA/PG-SGA, though comprehensive, largely depends on subjective clinician or patient assessment, which may introduce variability [25]. For sarcopenia, EWGSOP criteria produced a substantially lower prevalence than those using AWGS criteria. AWGS criteria use lower cutoff values for muscle mass and include a validated questionnaire for case finding, which may explain its higher detection rate than EWGSOP [26], [27]. Besides, the geographic distribution of studies using these criteria further complicates direct comparison. Thus, the marked variability in absolute estimates from different criteria underscores the urgent need for harmonized, IBD-specific diagnostic frameworks to enable accurate burden estimation, valid cross-regional comparisons, and the development of targeted interventions.

Exploratory geographic analyses revealed a distinct east-west divide, with the Western Pacific Region showing higher prevalence of underweight, malnutrition, and muscle depletion, whereas overweight and obesity dominated in the Region of the Americas and the European Region. However, these observed patterns should be interpreted primarily as exploratory, as they may be driven largely by methodological factors rather than the true geographic variation. Specifically, the diagnostic criteria for sarcopenia were unevenly distributed across regions, with AWGS criteria predominantly applied in Asian studies, yielding significantly higher estimates than EWGSOP criteria used in European cohorts. Similarly, the malnutrition estimate was disproportionately contributed by hospital-based studies from a single country (China), making it difficult to disentangle true regional differences from sampling artifacts. Thus, given these inherent confounding factors, the geographic patterns presented here require validation in future studies with standardized, population-based designs.

Our findings should be interpreted with consideration for their generalizability. An important consideration is the predominance of hospital-based studies, which comprised >80% of the included studies. Our subgroup analysis by study setting revealed that prevalence estimates for underweight, overweight, and obesity in population-based studies were broadly consistent with the main analyses, and similar demographic and clinical patterns persisted. However, population-based studies reporting malnutrition or sarcopenia were scarce, precluding robust subgroup analyses for these outcomes. Thus, although the consistency across settings for BMI-based outcomes suggests that our primary estimates are not substantially biased by hospital sampling, the generalizability of malnutrition and sarcopenia estimates to community-dwelling patients remains uncertain, highlighting the need for more population-based research using consensus-based diagnostic tools. Another concern is the marked sample size imbalance across outcomes. BMI-based estimates were derived from large cohorts, reflecting the widespread availability of BMI data, whereas sarcopenia estimates were based on far smaller samples because of the technical demands of muscle mass and function assessment. This smaller evidence base may affect the precision and generalizability of sarcopenia estimates. Encouragingly, emerging evidence suggests that muscle ultrasound—a noninvasive, bedside, and cost-effective tool—may facilitate wider screening for sarcopenia in IBD, with a recent study demonstrating good diagnostic accuracy and excellent interrater reliability compared with conventional methods [28].

Publication bias is another important consideration when interpreting the pooled estimates. For underweight and overweight, trim-and-fill adjustments yielded corrected estimates of 9.7% and 23.1%, respectively, closely aligned with the original pooled estimates (11.5% and 28.7%), suggesting that these results are relatively robust despite some funnel plot asymmetry. Nevertheless, the high heterogeneity across all outcomes suggests that clinical and methodological heterogeneity, rather than publication bias alone, likely contributes to funnel plot asymmetry. The uneven geographic distribution of included studies—the Western Pacific Region contributed disproportionately to the malnutrition estimates, and most of these studies came from a single country (China), which may amplify regional effects and bias the pooled estimate. These observations collectively underscore the need for larger, well-designed, population-based studies with standardized diagnostic criteria to provide more robust estimates.

Some limitations of this work should be acknowledged. First, the predominance of cross-sectional designs in the included studies precludes any causal inference and limits the assessment of temporal dynamics. Second, the substantial heterogeneity observed, even after extensive subgroup analyses, suggests unmeasured confounding from patient characteristics, treatment protocols, lifestyle factors, etc. For example, metabolic comorbidities might add complexity. It has been reported that metabolic syndrome affects ∼21.8% [29] and nonalcoholic fatty liver disease ≤30.7% of patients with IBD [30]. Diabetes has been demonstrated to be strongly associated with altered body composition, muscle loss, and chronic inflammation [31]. Thus, metabolic comorbidities may have an impact on the epidemiological estimates. Third, the generalizability of the pooled estimates should be considered. The geographic pattern at the regional and national level is constrained by the limited number of included studies in specific regions and countries, limiting the extent to which these estimates can be considered representative of the entire region or country. Furthermore, studies for malnutrition and sarcopenia were all hospital-based, typically enrolling patients with more active or complex disease, which may overestimate the true prevalence of these 2 nutritional disorders in the general IBD population. Fourth, our analysis did not capture micronutrient deficiencies, which represent another critical component of nutritional disorders in IBD. A recent large Italian multicenter study reported that 23% of patients with IBD had ≥1 micronutrient deficiency [32], highlighting that the burden of nutritional disorders extends beyond macronutrient alterations. Lastly, reliance on study-level data prevented multivariate adjustments for key confounders, such as disease activity and metabolic comorbidities. The subgroup ORs also reflect ecological comparisons between study-level subgroups, not within-study patient-level effects.

Notwithstanding these limitations, our findings provide a broad overview of the prevalence of major nutritional disorders in patients with IBD, highlighting that nutritional care in IBD should go beyond simple weight monitoring. However, given the wide PIs, these pooled estimates should be viewed as the overall level across highly heterogeneous settings rather than a precise global figure. Future research, particularly population-based studies with standardized diagnostic criteria across diverse regions, is needed to validate our observation.

## Author contributions

The authors’ responsibilities were as follows – SX, WX: conceived the study and participated in its design and coordination; TC, ZZ, MG: accessed to all data collection, analysis, and interpretation, and drafted the manuscript; YP, ZG: contributed to data collection and data analysis; SX, WX: critically revised the manuscript; and all authors: read and approved the final manuscript.

## Data availability

Only publicly available data were used in this study, and data sources and handling of these data are described in the Materials and methods and Supplemental Materials. Further information is available from the corresponding author on request.

## Ethics statement

Only publicly available data were used in this study, and there was no personally identifiable information; thus, informed consent was waived in this study.

## Declaration of Generative AI and AI-assisted technologies in the writing process

The authors declare that no generative AI or AI-assisted technologies were used in the writing of this manuscript.

## Funding

This study was supported by the Sichuan Science and Technology Program (2024NSFSC1687).

## Conflict of interest

The authors report no conflicts of interest.
