# Supplementary material for: The Prevalence of Nutritional Disorders in Inflammatory Bowel Disease: A Systematic Review and Meta-Analysis
Source: Adv Nutr. 2026 Oct 17;17(12):100758. doi: 10.1016/j.advnut.2026.100758 (PMC13769511; doi:10.1016/j.advnut.2026.100758)
Supplement: Supplementary file 1 [file mmc1.docx]

**The prevalence of nutritional disorders in inflammatory bowel disease: A systematic review and meta-analysis. Tong Chen, et al.**

**Supplementary materials**

**Appendixes**

**Appendix 1**. Search strategy

**Appendix 2**. Definition and diagnostic criteria of different nutritional disorders in patients with IBD

**Supplementary Tables**

**Supplementary Table 1**. Characteristics of included studies

**Supplementary Table 2.** Pooled analysis of malnutrition prevalence under ESPEN, GLIM and PGA/SGA criteria across subgroups

**Supplementary Table 3.** Pooled analysis of sarcopenia prevalence under EWGSOP and AWGS criteria across subgroups

**Supplementary Table 4.** Population-based prevalence of underweight I in IBD across subgroups

**Supplementary Table 5.** Population-based prevalence of overweight and obesity in IBD across subgroups

**Supplementary Table 6.** Prevalence of underweight in patients with IBD at the regional and country level

**Supplementary Table 7.** Prevalence of consensus-defined malnutrition in patients with IBD at the regional and country level

**Supplementary Table 8.** Prevalence of low muscle mass in patients with IBD at the regional and country level

**Supplementary Table 9.** Prevalence of sarcopenia in patients with IBD at the regional and country level

**Supplementary Table 10.** The odds ratios (ORs) for the prevalence of nutritional disorders in patients with inflammatory bowel disease grouped by WHO regions

**Supplementary Table 11.** Prevalence of overweight in patients with IBD at the reginal and country level

**Supplementary Table 12.** Prevalence of obesity in patients with IBD at the reginal and country level

**Supplementary Figures**

**Supplementary Figure 1.** Publication bias assessment of the pooled estimates of underweight in patients with inflammatory bowel disease.

**Supplementary Figure 2.** Publication bias assessment of the pooled estimates of consensus-defined malnutrition in patients with inflammatory bowel disease.

**Supplementary Figure 3.** Publication bias assessment of the pooled estimates of overweight and obesity in patients with inflammatory bowel disease.

**Supplementary Figure 4.** Forest plot of the prevalence of visceral obesity in patients with inflammatory bowel disease.

**Supplementary Figure 5.** Funnel plot of pooled prevalence of low muscle mass in patients with inflammatory bowel disease.

**Supplementary Figure 6.** Pooled prevalence rate of low muscle mass with obesity in patients with inflammatory bowel disease.

**Supplementary Figure 7.** Pooled prevalence rate of malnutrition grouped by consensus criteria (GLIM, ESPEN, MNA and SGA/PG-SGA).

**Supplementary Figure 8.** Forest plot of the prevalence of malnutrition based on ICD coding.

**Supplementary Figure 9.** Forest plot of the pooled prevalence of sarcopenia grouped by specific criteria.

**Appendix 1. Search strategy**

PubMed (n=7479)

#1. Inflammatory Bowel Diseases[MeSH Terms]

#2. inflammatory bowel disease[Title/Abstract] OR crohn’s disease[Title/Abstract] OR ulcerative colitis[Title/Abstract] OR IBD[Title/Abstract] OR CD[Title/Abstract] OR UC[Title/Abstract]

#3. #1 OR #2

#4. Malnutrition[MeSH Terms]

#5. malnutrition[Title/Abstract] OR sarcopenia[Title/Abstract] OR overweight[Title/Abstract] OR obesity[Title/Abstract] OR micronutrient deficienc*[Title/Abstract] OR protein energy malnutrition[Title/Abstract] OR underweight[Title/Abstract] OR emaciation[Title/Abstract]

#6. #4 OR #5

#7. #3 AND #6

Cochrane Library (n=826)

#1	MeSH descriptor: [Inflammatory Bowel Diseases] explode all trees

#2	(Crohn’s disease or ulcerative colitis or IBD or CD or UC):ti,ab,kw (Word variations have been searched)

#3	#1 or #2

#4	MeSH descriptor: [Malnutrition] explode all trees

#5	(sarcopenia or overweight or obesity or micronutrient deficiencies or protein-energy malnutrition or underweight or emaciation):ti,ab,kw (Word variations have been searched)

#6	#4 or #5

#7	#3 and #6

Embase (n=9841)

#1. 'Inflammatory Bowel Diseases'/exp

#2**.** 'inflammatory bowel disease':ti,ab,kw OR 'crohn’s disease':ti,ab,kw OR 'ulcerative colitis':ti,ab,kw OR 'IBD':ti,ab,kw OR 'CD':ti,ab,kw OR 'UC':ti,ab,kw

#3. #1 OR #2

#4. 'Malnutrition'/exp

#5. 'malnutrition':ti,ab,kw OR 'sarcopenia':ti,ab,kw OR 'overweight':ti,ab,kw OR 'obesity':ti,ab,kw OR 'micronutrient deficienc*':ti,ab,kw OR 'protein energy malnutrition':ti,ab,kw OR 'underweight':ti,ab,kw OR 'emaciation':ti,ab,kw

#6. #4 OR #5

#7. #3 AND #6

CBM(n=736)

#1 "Inflammatory Bowel Diseases"[Common tags: Automatic] OR "crohn’s disease"[Common tags: Automatic] OR "ulcerative colitis"[Common tags: Automatic]

#2 "malnutrition" [Common tags: Automatic] OR "sarcopenia" [Common tags: Automatic] OR "obesity" [Common tags: Automatic] OR "overweight"[Common tags: Automatic] OR "emaciation" [Common tags: Automatic] OR "underweight" [Common tags: Automatic] OR "micronutrient deficiencies" [Common tags: Automatic] OR "protein energy malnutrition" [Common tags: Automatic]

#3 #1 AND #2

**Appendix 2. Definition and diagnostic criteria of different nutritional disorders in patients with IBD**

**Underweight**

Underweight was defined using established age-specific thresholds. For adults, underweight was defined as BMI < 18.5 kg/m² according to World Health Organization (WHO) criteria. For children and adolescents, given that normal BMI varies with age and sex during growth, underweight was defined as BMI-for-age more than 1 standard deviation below the WHO Growth Reference median for those aged 5-19 years, or weight-for-height more than 2 standard deviations below the WHO Child Growth Standards median for children under 5 years. For studies using national growth charts, age- and sex-specific BMI below the 3th or 5th percentile was also accepted.

**Consensus-based malnutrition**

Malnutrition is defined as a state of nutrition in which a deficiency or imbalance of energy, protein, and other nutrients causes measurable adverse effects on tissue/body form, function, and clinical outcome(1). To ensure comprehensive inclusion of relevant studies, this review considered a study eligible if it diagnosed malnutrition using any of the following consensus criteria or tools: Global Leadership Initiative on Malnutrition (GLIM) criteria(2), European Society for Clinical Nutrition and Metabolism (ESPEN) diagnostic criteria(3), Subjective Global Assessment (SGA) or its variant Patient-Generated SGA (PG-SGA)(4, 5), Mini Nutritional Assessment (MNA) criteria(6), or ICD (Internation Code of Disease) codes for protein-energy malnutrition.

**Overweight, obesity and visceral obesity**

For whole-body adiposity, overweight or obesity is classified using Body Mass Index (BMI) based on either the WHO International standard or the Asian-specific BMI cut-off values. The WHO defines overweight as a BMI between 25 and 30 kg/m² and obesity as a BMI ≥ 30.0 kg/m². For Asian populations, the accepted cut-offs for overweight are defined as BMI between 23.0 and 25.0 kg/m² and obesity as BMI ≥ 25.0 kg/m²(7). Regarding the diagnostic criteria for visceral obesity, established anthropometric measures such as waist circumference or, more definitively, by imaging techniques like computed tomography (CT) which directly quantifies visceral fat area were accepted. Thus, studies reporting the prevalence of either general obesity (by BMI) or visceral obesity (by the aforementioned criteria) were included in our synthesis.

**Muscle depletion**

Muscle depletion in IBD encompasses a spectrum of body composition abnormalities, ranging from isolated loss of muscle mass to more functionally significant impairment. A substantial body of literature has focused on low muscle mass, defined solely by a reduced Skeletal Muscle Index (SMI) derived from cross-sectional imaging such as CT or MRI-a measure that captures anatomical deficit without considering functional performance(8). An alternative and clinically more stringent construct is consensus-based sarcopenia, which requires not only low muscle mass but also the presence of impaired muscle function (e.g., low grip strength or reduced physical performance), as defined by the European Working Group on Sarcopenia in Older People (EWGSOP)(9, 10) and the Asian Working Group for Sarcopenia (AWGS)(11, 12). Additionally, we examined the overlap of muscle depletion with excess adiposity-specifically, low muscle mass with obesity (assessed by SMI plus BMI-based obesity) and sarcopenic obesity (consensus-based sarcopenia plus obesity)-to address this sub-phenotype within the muscle depletion spectrum.

**Reference**

1.	Cederholm T, Barazzoni R, Austin P, Ballmer P, Biolo G, Bischoff SC et al. ESPEN guidelines on definitions and terminology of clinical nutrition. *Clinical Nutrition*. 2017;36(1):49-64.

2.	Cederholm T, Jensen GL, Correia M, Gonzalez MC, Fukushima R, Higashiguchi T et al. GLIM criteria for the diagnosis of malnutrition - A consensus report from the global clinical nutrition community. *Clinical nutrition (Edinburgh, Scotland)*. 2019 Feb;38(1):1-9.

3.	Cederholm T, Bosaeus I, Barazzoni R, Bauer J, Van Gossum A, Klek S et al. Diagnostic criteria for malnutrition - An ESPEN Consensus Statement. *Clinical nutrition (Edinburgh, Scotland)*. 2015 Jun;34(3):335-40.

4.	Bauer J, Capra S, Ferguson M. Use of the scored Patient-Generated Subjective Global Assessment (PG-SGA) as a nutrition assessment tool in patients with cancer. *Eur J Clin Nutr*. 2002 Aug;56(8):779-85.

5.	Muscaritoli M, Arends J, Bachmann P, Baracos V, Barthelemy N, Bertz H et al. ESPEN practical guideline: Clinical Nutrition in cancer. *Clinical nutrition (Edinburgh, Scotland)*. 2021 May;40(5):2898-913.

6.	Vellas B, Villars H, Abellan G, Soto ME, Rolland Y, Guigoz Y et al. Overview of the MNA--Its history and challenges. *The journal of nutrition, health & aging*. 2006 Nov-Dec;10(6):456-63; discussion 63-5.

7.	Consultation WE. Appropriate body-mass index for Asian populations and its implications for policy and intervention strategies. *Lancet*. 2004 Jan 10;363(9403):157-63.

8.	Martin L, Birdsell L, Macdonald N, Reiman T, Clandinin MT, McCargar LJ et al. Cancer cachexia in the age of obesity: skeletal muscle depletion is a powerful prognostic factor, independent of body mass index. *J Clin Oncol*. 2013 Apr 20;31(12):1539-47.

9.	Cruz-Jentoft AJ, Baeyens JP, Bauer JM, Boirie Y, Cederholm T, Landi F et al. Sarcopenia: European consensus on definition and diagnosis: Report of the European Working Group on Sarcopenia in Older People. *Age and ageing*. 2010 Jul;39(4):412-23.

10.	Cruz-Jentoft AJ, Bahat G, Bauer J, Boirie Y, Bruyère O, Cederholm T et al. Sarcopenia: revised European consensus on definition and diagnosis. *Age and ageing*. 2019 Jan 1;48(1):16-31.

11.	Chen LK, Liu LK, Woo J, Assantachai P, Auyeung TW, Bahyah KS et al. Sarcopenia in Asia: consensus report of the Asian Working Group for Sarcopenia. *Journal of the American Medical Directors Association*. 2014 Feb;15(2):95-101.

12.	Chen LK, Woo J, Assantachai P, Auyeung TW, Chou MY, Iijima K et al. Asian Working Group for Sarcopenia: 2019 Consensus Update on Sarcopenia Diagnosis and Treatment. *Journal of the American Medical Directors Association*. 2020 Mar;21(3):300-7.e2.

**Supplementary Tables**

**Supplementary Table 1. Characteristics of included studies**

| Author (year) | **WHO Region** | **Population-based setting** | **Study period** | **No. Patients** | **Gender subgroup** | **Population group** | **IBD subtype** | **Disease activity** | **Nutritional Disorders (Criteria)** | **Quality Score** |
| --- | --- | --- | --- | --- | --- | --- | --- | --- | --- | --- |
| Blain (2002)(1) | European Region | YES | 1974-2000.12 | 2065 | YES | Mixed | CD | NO | Obesity (BMI) | 7 |
| Hass (2006)(2) | Region of the Americas | NO | 1997-2002 | 148 | YES | NA | CD | NO | Overweight or Obesity (BMI),  Underweight (BMI) | 8 |
| Nguyen (2008)(3) | Region of the Americas | YES | 1998-2004 | 52142 | NO | Adult | CD UC | NO | PCM (ICD) | 8 |
| Gao (2009)(4) | Western Pacific Region | NO | 2008.1-2009.1 | 112 | NO | Mixed | CD UC | NO | Underweight (BMI) | 6 |
| Steed (2009)(5) | European Region | NO | NA | 489 | NO | NA | CD UC | NO | Obesity (BMI), Overweight (BMI),  Underweight (BMI) | 5 |
| Rocha (2009)(6) | Region of the Americas | NO | 2005.8-2006.9 | 102 | NO | Adult | CD UC | YES | Underweight (BMI) | 5 |
| Vasseur (2010)(7) | European Region | NO | 1988-2004 | 261 | NO | Children or adolescent | CD | NO | Underweight (WHO) | 8 |
| Long (2011)(8) | Region of the Americas | YES | 2007.4.1-2009.12.31 | 1598 | YES | Children or adolescent | CD UC | NO | Overweight or obesity (BMI) | 9 |
| Causey (2011)(9) | Region of the Americas | YES | 2005-2008 | 2319 | YES | Adult | CD | NO | Obesity (BMI) | 7 |
| Guo (2013)(10) | Western Pacific Region | NO | 2009.1-2012.1 | 130 | NO | Mixed | UC | NO | Underweight (BMI) | 6 |
| Zwintscher (2014)(11) | European Region | YES | 2009.1-2009.12 | 12465 | YES | Children or adolescent | CD UC | NO | Obesity (BMI) | 7 |
| Stabroth-Akil (2015)(12) | European Region | NO | 2000-2006 | 202 | YES | Adult | UC | NO | Obesity (BMI), Overweight (BMI),  Underweight (BMI) | 7 |
| Seminerio (2015)(13) | Region of the Americas | YES | 2009-2011 | 1494 | YES | Adult | CD UC | NO | Obesity (BMI), Overweight (BMI),  Underweight (BMI) | 8 |
| Flores (2015)(14) | Region of the Americas | NO | 2000.1-2012.12 | 581 | YES | Adult | CD UC | NO | Obesity (BMI), Overweight (BMI) | 8 |
| Manne (2015)(15) | Region of the Americas | NO | After 2010 | 120 | NO | Adult | CD UC | NO | Overweight (BMI), Obesity (BMI) | 7 |
| Dotson (2015)(16) | Region of the Americas | YES | 2004.4-2012.6 | 5782 | NO | Children or adolescent | CD | NO | Malnutrition (ICD) | 7 |
| Bryant (2015)(17) | Western Pacific Region | NO | 2012.4-2013.7 | 137 | YES | Adult | CD UC | NO | Sarcopenia (EWGSOP) | 8 |
| Brown (2016)(18) | European Region | NO | 1999-2012 | 388 | NO | Mixed | CD | NO | Overweight (BMI), Obesity (BMI) | 9 |
| Raffner Basson (2016)(19) | South Africa | NO | 2011.09-2013.01 | 186 | NO | Adult | CD | NO | Overweight or obesity (BMI) | 9 |
| Pituch-Zdanowska (2016)(20) | European Region | NO | 2005-2013 | 675 | NO | Children or adolescent | CD UC | NO | Obesity (BMI), Overweight (BMI) | 7 |
| Guardado (2016)(21) | Region of the America | NO | 2000-2014 | 391 | YES | Adult | CD UC | NO | Obesity (BMI), Overweight (BMI),  Underweight (BMI) | 7 |
| Frigstad (2017)(22) | European Region | NO | 2013.3-2014.4 | 408 | NO | Mixed | CD UC | NO | Overweight or obesity (BMI) | 7 |
| Casanova (2017)(23) | European Region | NO | 2015.1-2016.4 | 333 | NO | Adult | CD UC | NO | Overweight, obesity | 9 |
| Glassner (2017)(24) | Region of the America | NO | 2015.1-2016.4 | 112 | NO | Adult | CD UC | NO | Obesity (BMI) | 5 |
| Zhang (2017)(25) | Western Pacific Region | NO | 2011.5-2014.3 | 114 | YES | Adult | CD | NO | Low muscle mass (SMI) | 7 |
| Singla (2017)(26) | European Region | NO | 2011.6-2013.6 | 209 | YES | Adult | CD | NO | Obesity (BMI), Overweight (BMI)  Underweight (BMI) | 7 |
| Pallav (2017)(27) | Region of the Americas | NO | 2012.7-2015.7 | 211 | NO | NA | CD UC | NO | Obesity (BMI), Overweight (BMI) | 7 |
| Pedersen (2017)(28) | Region of the Americas | NO | 2010-2015 | 178 | NO | Adult | CD UC | NO | Obesity (BMI), Overweight (BMI) | 8 |
| Guerbau (2017)(29) | European Region | NO | 2010.1-2014.5 | 140 | YES | Adult | CD | NO | Obesity (BMI), Overweight (BMI) | 8 |
| Back (2017)(30) | Region of the Americas | NO | 2012.3-2012.12 | 141 | NO | Adult | CD UC | NO | Obesity (BMI), Overweight (BMI), Underweight (BMI) | 7 |
| Bryant (2018)(31) | Western Pacific Region | NO | 2012.4-2013.9 | 129 | NO | Adult | CD UC | NO | Obesity (BMI), Overweight (BMI), Underweight (BMI) | 8 |
| Chandradas (2018)(32) | Region of the Americas | NO | 2014.1.1-2015.12.31 | 636 | NO | Adult | UC | NO | Overweight (BMI), Obesity (BMI) | 7 |
| Jain (2018)(33) | Region of the Americas | NO | 2006.9-2014.10 | 898 | NO | Children or adolescent | CD | YES | Obesity (BMI), Overweight (BMI) | 7 |
| Ladd (2018)(34) | Region of the Americas | NO | 2012-2015 | 516 | NO | Children or adolescent | CD | NO | Malnutrition (WHO) | 9 |
| Madsen (2018)(35) | European Region | NO | 2003.1.1-2015.12.31 | 210 | NO | Adult | CD UC | NO | Overweight (BMI), Obesity (BMI) | 8 |
| Spooren (2019)(36) | European Region | NO | NA | 417 | NO | Adult | CD UC | NO | Obesity (BMI), Overweight (BMI), Underweight (BMI) | 8 |
| Marcil (2019)(37) | Region of the Americas | NO | 2008-2010 | 183, 371 | YES | Children or adolescent, Adult | CD UC | NO | Malnutrition BMI) | 8 |
| Lomer (2019)(38) | European Region | NO | NA | 390 | NO | Adult | CD UC | NO | Obesity (BMI), Overweight (BMI), Underweight (BMI) | 5 |
| Magrì (2019)(39) | European Region | NO | 2016.12-2018.1 | 178 | NO | Adult | CD UC | NO | Overweight (BMI), Obesity (BMI) | 9 |
| Qiao (2019)(40) | Western Pacific Region | NO | 2015.1-2015.12 | 198 | NO | Adult | CD UC | NO | Underweight (BMI), Overweight or obesity (BMI) | 6 |
| Nguyen (2019)(41) | Region of the Americas | YES | 2013.1-2013.6 | 42285 | NO | Adult | CD UC | NO | Obesity (ICD) | 7 |
| Mentella (2019)(42) | European Region | NO | 2016.10-2018.9 | 206 | NO | Adult | CD UC | NO | Obesity (BMI), Overweight (BMI), Underweight (BMI) | 7 |
| McKenna (2019)(43) | Region of the Americas | NO | 2007.1-2017.12 | 758 | YES | Adult | CD | NO | Obesity (BMI), Overweight (BMI), Underweight (BMI) | 8 |
| Jain (2019)(44) | Region of the Americas | YES | NA | 7296 | YES | Adult | CD UC | NO | Obesity (BMI), Overweight (BMI) | 8 |
| Pizzoferrato (2019)(45) | European Region | NO | 2017.2-2017.6 | 127 | NO | Adult | CD UC | NO | Sarcopenia (ASMI/SMI) | 5 |
| Shrestha (2019)(46) | Region of the Americas | YES | 2010-2014 | 12517 | NO | Adult | CD UC | NO | Obesity (BMI) | 8 |
| Chandrakumar (2020)(47) | Region of the Americans | NO | 2012.1-2018.8 | 139 | NO | Children or adolescent | CD UC | NO | Obesity (BMI) | 8 |
| Sun (2020)(48) | Western Pacific Region | NO | 2016.10-2018.3 | 135 | NO | Adult | CD UC | NO | Malnutrition (SGA/PG-SGA) | 8 |
| Kang (2020)(49) | Western Pacific Region | NO | 2004.1-2017.12 | 443 | NO | Adult | CD UC | NO | Obesity (BMI) | 8 |
| Zeng (2020)(50) | Western Pacific Region | NO | 2018.8-2019.10 | 188 | YES | Adult | CD | YES | Sarcopenia (EWGSOP), Underweight (BMI) | 7 |
| Labarthe (2020)(51) | European Region | NO | 2016.1-2019.1 | 132 | NO | Adult | CD | NO | Overweight (BMI), Underweight (BMI), Low muscle mass (SMI) | 9 |
| Greuter (2020)(52) | European Region | YES | 2006-2018 | 2050 | YES | Adult | CD UC | NO | Obesity (BMI) | 7 |
| EI Mouzan (2020)(53) | Eastern Mediterranean Region | NO | 2003-2012 | 374 | NO | Children or adolescent | CD UC | NO | Overweight (BMI), Underweight (BMI) | 8 |
| Cioffi (2020)(54) | European Region | NO | 2016.7-2018.3 | 140 | YES | Adult | CD | NO | Obesity (BMI), Overweight (BMI), Underweight (BMI) | 9 |
| Sun (2020)(55) | Western Pacific Region | NO | 2018.8-2019.6 | 138 | NO | Adult | CD | NO | Underweight (BMI) | 7 |
| Pulley (2020)(56) | Western Pacific Region | NA | NA | 107 | N | Adult | CD UC | NO | Malnutrition (SGA/PG-SGA, BMI),  Overweight or obesity (BMI) | 7 |
| Yerushalmy-Feler (2021)(57) | European Region | NO | 2010.1-2018.12 | 152 | NO | Children or adolescent | CD UC | NO | Overweight or obesity (WHO)  Underweight (WHO) | 8 |
| Ünal (2021)(58) | European Region | NO | 2019.3-2019.8 | 344 | YES | Adult | CD UC | YES | Obesity (BMI), Overweight (BMI), Malnutrition (MNA), Sarcopenia (EWGSOP2) | 7 |
| Prieto (2021)(59) | Region of the Americas | NO | 2012.1-2012.12 | 217 | NO | Adult | CD | YES | Obesity (BMI), Overweight (BMI), Underweight (BMI) | 7 |
| Johnson (2021)(60) | Region of the Americas | NO | 1970-2010 | 417 | YES | Adult | UC | NO | Obesity (BMI), Overweight (BMI), Underweight (BMI) | 7 |
| Kim (2021)(61) | Western Pacific Region | YES | 1989.6-2016.12 | 6127 | NO | Adult | CD UC | NO | Obesity (BMI) | 7 |
| Bian (2021)(62) | Western Pacific Region | NO | 2019.2-2019.6 | 146 | NO | Adult | CD | YES | Malnutrition (SGA/PG-SGA), Sarcopenia (AWGS) | 8 |
| Weissman (2021)(63) | Region of the Americas | YES | 2016-2017 | 143190 | YES | Adult | CD UC | NO | Obesity (BMI) | 8 |
| Wan (2021)(64) | Western Pacific Region | NO | 2016.7-2019.1 | 150 | NO | Adult | CD UC | NO | Visceral obesity (VAT/SAT) | 7 |
| Singh (2021)(65) | Region of the Americas | YES | 2014-2018 | 5987 | YES | Adult | CD UC | NO | Obesity (ICD) | 8 |
| Taylor (2021)(66) | Region of the Americas | NO | 2017.5-2018.3 | 245 | NO | Adult | CD UC | NO | Malnutrition (SGA), Overweight or obesity (BMI) | 10 |
| Marino (2021)(67) | European Region | NO | 2019.11-2020.2,  2020.7-2020.11 | 116 | NO | Children or adolescent | CD UC | NO | Malnutrition (WHO), Overweight (WHO) | 7 |
| Park (2021)(68) | Region of the Americas | YES | After 2010 | 7272 | YES | Adult | CD UC | NO | Obesity (BMI) | 7 |
| EI-Dallal (2021)(69) | Region of the Americas | YES | 2016 | 61075 | YES | Adult | UC | NO | Obesity (ICD/BMI) | 7 |
| Egberg (2021)(70) | Region of the Americas | YES | 2012-2018 | 1258 | NO | Children or adolescent | CD | NO | Obesity (WHO), Overweight (WHO), Malnutrition (WHO) | 8 |
| Vohra (2021)(71) | Region of the Americas | YES | 2016-2017 | 50046 | NO | Adult | UC | NO | Obesity (BMI) | 9 |
| Zhang (2021)(72) | Western Pacific Region | NO | 2013.1-2019.12 | 124 | YES | Adult | CD | NO | Low muscle mass (SMI), Underweight (BMI), Overweight (BMI) | 7 |
| Chuck (2022)(73) | Western Pacific Region | NO | 2016-2019 | 181 | NO | Adult | UC CD | NO | Obesity (BMI) | 8 |
| Shen (2022)(74) | Western Pacific Region | NO | 2019.11-2021.11 | 104 | YES | Adult | UC | NO | Malnutrition (GLIM) | 9 |
| Trinder (2022)(75) | Western Pacific Region | NO | 2010.1-2020.12 | 147 | YES | Adult | CD UC | NO | Low muscle mass (SMI) | 7 |
| Liu (2022)(76) | Western Pacific Region | YES | 2017.7-2017.12 | 1013 | YES | Adult | CD UC | YES | Overweight or obesity (BMI), Malnutrition (ESPEN, BMI) | 9 |
| Johnson (2022)(77) | Region of the Americas | NO | 1970-2010 | 334 | YES | Adult | CD | NO | Obesity (BMI), Overweight (BMI), Underweight (BMI) | 8 |
| Gordon (2022)(78) | Region of the Americas | NO | 2014-2019 | 291 | NO | Adult | CD UC | NO | Obesity (BMI) | 8 |
| Abd-El-aziz (2022)(79) | Region of the Americas | YES | 2005-2018 | 25431 | YES | Adult | CD UC | NO | Malnutrition (ESPEN) | 7 |
| Aljohani (2022)(80) | Eastern Mediterranean Region | NO | 2009.1.1-2019.12.31 | 435 | NO | Mixed | CD UC | NO | Underweight (BMI), Overweight (BMI), Obesity (BMI) | 8 |
| Yelencich (2022)(81) | Region of the Americas | NO | 2019.10-2020.3 | 161 | NO | Adult | CD UC | NO | Obesity (BMI), Overweight (BMI),  Malnutrition (SGA/PGA-SGA) | 8 |
| Zhang (2022)(82) | Western Pacific Region | NO | 2020.9-2021.5 | 238 | NO | Adult | CD UC | NO | Malnutrition (GLIM/SGA/ESPEN/BMI), Overweight (BMI), Obesity (BMI) | 10 |
| Xu (2022)(83) | Western Pacific Region | NO | 2020.5-2021.2 | 220 | NO | Adult | UC | NO | Overweight or obesity (BMI), Underweight (BMI) | 9 |
| von Graffenried (2022)(84) | European Region | NO | 2006.5-2020.5 | 327 | YES | Children or adolescent | CD UC | NO | Obesity (WHO), Overweight (WHO), Malnutrition (WHO) | 7 |
| Tseng (2022)(85) | Western Pacific Region | NO | 2016.1-2022.3 | 167 | YES | Adult | CD | NO | Low muscle mass (SMI) | 8 |
| Peng (2022)(86) | Western Pacific Region | NO | 2021.5-2022.2 | 169 | YES | Adult | CD | YES | Malnutrition (GLIM) | 8 |
| Reiner (2022)(87) | European Region | NO | 2018.12-2019.10 | 101 | YES | Mixed | CD UC | NO | Malnutrition (GLIM/ESPEN/BMI), Overweight or obesity (BMI) | 9 |
| Liu (2022)(88) | Western Pacific Region | NO | 2020.9-2021.9 | 110 | YES | Adult | CD UC | NO | Sarcopenia (AWGS), Sarcopenic obesity (sarcopenia with body fat percentage), \Low muscle mass (SMI) | 8 |
| Ohya (2022)(89) | Western Pacific Region | NO | 2013.1-2019.10 | 101 | NO | Adult | UC | NO | Overweight or obesity (BMI) | 6 |
| Kuloglu (2022)(90) | European Region | NO | 1998-2016 | 824 | NO | Children or adolescent | CD UC | NO | Malnutrition (WHO), Overweight (BMI) | 9 |
| Li (2022)(91) | Western Pacific Region | NO | 2020.3-2021.3 | 108 | NO | Adult | CD UC | NO | Malnutrition (GLIM) | 6 |
| Karachaliou (2022)(92) | European Region | NO | 2018.11-2019.11 | 237 | NO | Adult | CD | YES | Obesity (BMI), Overweight (BMI),  Malnutrition (GLIM), Sarcopenia (EWGSO) | 7 |
| Kadenczki (2022)(93) | European Region | YES | 2010-2016 | 1027 | NO | Children or adolescent | CD UC | NO | Obesity (WHO), Malnutrition (WHO) | 7 |
| Jin (2022)(94) | Western Pacific Region | NO | 1995.1-2018.1 | 105 | YES | Children or adolescent | CD | NO | Malnutrition (WHO) | 7 |
| Hoffman (2022)(95) | Region of the Americas | YES | 2015.1-2020.1 | 263100 | NO | Adult | CD UC | NO | Obesity (BMI) | 7 |
| Gu (2022)(96) | Region of the Americas | YES | 2010.1-2017.6 | 3038 | YES | Adult | CD UC | NO | Obesity (BMI), Overweight (BMI) | 8 |
| Jain (2022)(97) | Region of the Americas | YES | 2006.9-2018.12 | 4972 | NO | Children or adolescent | CD UC | YES | Overweight (BMI), Obesity (BMI) | 8 |
| Ge (2022)(98) | Western Pacific Region | NO | 2015.12-2020.3 | 254 | YES | Adult | UC | NO | Obesity (BMI), Overweight (BMI), Underweight (BMI), Low muscle mass (SMI) | 7 |
| Dahiya (2022)(99) | Region of the Americas | YES | 2016-2018 | 282005 | YES | Adult | CD UC | NO | Obesity (BMI), Malnutrition (ICD) | 7 |
| Campbell (2022)(100) | Region of the Americas | NO | 2005-2018 | 156 | NO | Adult | CD UC | NO | Low muscle mass (SMI) | 7 |
| Shehab (2022)(101) | Eastern Mediterranean Region | NO | 2021.1-2021.11 | 528 | NO | Adult | CD UC | NO | Obesity (BMI), Overweight (BMI), Underweight (BMI) | 8 |
| Wang (2022)(102) | Western Pacific Region | NO | 2016.8-2019.12 | 146 | NO | Adult | CD | NO | Malnutrition (SGA/PG-SGA) | 6 |
| Abu-Freha (2023)(103) | European Region | YES | 2000-2021 | 25269 | NO | Mixed | CD UC | NO | Obesity (BMI) | 7 |
| Wei (2023)(104) | Western Pacific Region | NO | 2014.8-2022.8 | 605 | YES | Adult | UC | YES | Malnutrition (GLIM), Overweight (BMI), Obesity (BMI) | 8 |
| Wang (2023)(105) | Western Pacific Region | NO | 2021.3-2021.8 | 188 | YES | Adult | CD UC | YES | Malnutrition (GLIM) | 9 |
| Singh (2023)(106) | South East Asia | NO | 2020.10-2021.6 | 406 | YES | Adult | CD UC | YES | Malnutrition (ESPEN) | 7 |
| Tang (2023)(107) | Western Pacific Region | NO | 2019.12-2023.4 | 106 | NO | Mixed | CD UC | NO | Underweight (BMI), Overweight or obesity (BMI), Pre-sarcopenia, Visceral obesity | 10 |
| Sim (2023)(108) | Western Pacific Region | NO | 2018.3-2018.6 | 217 | NO | Adult | CD UC | NO | Overweight or obesity (BMI), Underweight (BMI) | 8 |
| Sehgal (2023)(109) | Region of the Americas | YES | 2020-2021 | 2695 | NO | Adult | CD UC | NO | Obesity (BMI) | 7 |
| Gold (2023)(110) | Region of the Americas | NO | NA | 182 | YES | Adult | CD UC | NO | Obesity (BMI), Overweight (BMI), Malnutrition (ESPEN) | 7 |
| Elangovan (2023)(111) | Region of the Americas | YES | 2010-2019 | 286760 | YES | Mixed | CD UC | NO | Obesity (BMI) | 7 |
| Fernandez (2023)(112) | Region of the Americas | YES | 2005-2019 | 49746 | NO | Adult | CD UC | NO | Underweight (BMI), Overweight (BMI), Obesity (BMI) | 9 |
| Dua (2023)(113) | Region of the Americas | YES | 2010-2018 | 2048964 | YES | Mixed | CD UC | NO | PCM (ICD) | 7 |
| Bacha (2023)(114) | European Region | YES | 2018.11.26-11.30 | 1704 | YES | Adult | CD UC | NO | Obesity (BMI), Overweight (BMI), Underweight (BMI) | 8 |
| Zhang (2023)(115) | Western Pacific Region | NO | 2020.1-2022.7 | 116 | NO | Adult | UC | YES | Underweight (BMI) | 8 |
| Wang (2023)(116) | Western Pacific Region | NO | 2020.12-2022.02 | 115 | NO | Adult | CD | NO | Malnutrition (MNA) | 7 |
| Wang (2023)(117) | Region of the Americas | YES | 2018.01-06 | 63748 | NO | Adult | CD UC | NO | Overweight or obesity (BMI) | 8 |
| Nam (2023)(118) | Western Pacific Region | NO | 1989.6-2016.12 | 1027 | NO | Mixed | CD UC | NO | Overweight or obesity (BMI), Low muscle mass (SMI), Sarcopenic obesity (SMI+BMI) | 7 |
| Grova (2023)(119) | European Region | NO | 2014-2020 | 358 | YES | Adult | CD | NO | Low muscle mass (SMI) | 8 |
| Cankurtaran (2023)(120) | European Region | NO | 2015.1-2021.08 | 116 | YES | Adult | CD | NO | Low muscle mass (SMI) | 7 |
| Barajas Ordonez (2023)(121) | European Region | NO | 2010.6-2020.4 | 114 | NO | Adult | CD | NO | Overweight or obesity (BMI),  Low muscle mass (SMI), Underweight (BMI) | 7 |
| Barnes (2023)(122) | Western Pacific Region | NO | NA | 670 | NO | Adult | CD UC | NO | Obesity (BMI) | 5 |
| Bedard (2023)(123) | Region of the Americas | NO | 2017.5-2018.3 | 161 | NO | Adult | CD UC | NO | Obesity (BMI) | 7 |
| Choi (2023)(124) | Western Pacific Region | NO | 2009.1-2021.8 | 112 | YES | Adult | CD | NO | Sarcopenia (BMI) | 8 |
| Cioffi (2023)(125) | European Region | NO | 2016.7-2018.3 | 140 | NO | Adult | CD | NO | Obesity (BMI), Overweight (BMI), Underweight (BMI) | 8 |
| García-Mateo (2023)(126) | European Region | NO | 2019.10-2020.4 | 688 | YES | Adult | CD UC | NO | Obesity (BMI), Overweight (BMI), Underweight (BMI) | 9 |
| Huang (2023)(127) | Western Pacific Region | NO | 2020.9-2021.5 | 177 | YES | Adult | CD | NO | Malnutrition (GLIM) | 8 |
| Kim (2023)(128) | Western Pacific Region | NO | 1995.1-2020.12 | 165 | NO | Adult | CD | NO | Obesity (BMI), Overweight (BMI), Underweight (BMI) | 8 |
| Lee (2023)(129) | Western Pacific Region | NO | 2010.1-2016.12 | 9855 | NO | Adult | CD UC | NO | Obesity (BMI) | 8 |
| Lima (2023)(130) | Region of the Americas | NO | 2021.1-2021.12 | 470 | YES | Adult | CD UC | NO | Obesity (BMI), Overweight (BMI), Underweight (BMI) | 6 |
| Traiki (2023)(131) | Eastern Mediterranean Region | NO | 2010.1-2018.10 | 169 | NO | NA | CD | NO | Underweight (BMI) | 7 |
| Wall (2023)(132) | European Region | NO | 2018-2020 | 282 | NO | Adult | CD UC | NO | Underweight (BMI) | 9 |
| Yoon (2023)(133) | Western Pacific Region | NO | 2014.8-2017.2 | 211 | NO | Mixed | UC | YES | Obesity (BMI), Overweight (BMI), Underweight (BMI) | 8 |
| Zhang (2023)(134) | Western Pacific Region | NO | 2020.9-2021.5 | 238 | NO | Adult | CD UC | YES | Malnutrition (GLIM), Sarcopenia (AWGS) | 7 |
| Zhi (2023)(135) | Western Pacific Region | NO | 2013.1-2020.8 | 191 | YES | Mixed | CD | NO | Low muscle mass (SMI) | 8 |
| Zhou (2023)(136) | Western Pacific Region | NO | 2015.1-2021.12 | 125 | NO | Children or adolescent | CD UC | NO | Malnutrition (WHO) | 8 |
| Shen (2023)(137) | Western Pacific Region | NO | 2018.1-2022.7 | 105 | YES | Adult | CD | YES | Low muscle mass (SMI), Underweight (BMI) | 7 |
| Zhou (2023)(138) | Western Pacific Region | NO | 2018.1-2023.2 | 121 | YES | Adult | CD | YES | Low muscle mass (SMI) | 8 |
| Algin (2024)(139) | European Region | NO | 2017-2022 | 112 | NO | Adult | CD | NO | Underweight (BMI), Overweight (BMI), Obesity (BMI) | 5 |
| Desai (2024)(140) | Region of the Americas | YES | 2010.1.1-2022.12.31 | 11629 | NO | Adult | CD UC | NO | Overweight (BMI), Obesity (BMI) | 8 |
| Ebach (2024)(141) | Region of the Americas | NO | NA | 224 | YES | Children or adolescent | CD | NO | Overweight or obesity (BMI) | 7 |
| Ergenc (2024)(142) | European Region | NO | 2020.1-2020.9 | 129 | NO | Adult | CD UC | NO | Sarcopenia (EWGSOP) | 9 |
| Hu (2024)(143) | Western Pacific Region | NO | 2019.7-2021.12 | 212 | YES | Adult | CD | NO | Low muscle mass (SMI) | 8 |
| Kodia (2024)(144) | Region of the Americas | YES | 2016-2020 | 1464 | NO | Adult | CD | NO | Underweight (BMI) | 7 |
| Li (2024)(145) | Western Pacific Region | NO | 2015.6-2021.12 | 118 | NO | Mixed | CD | NO | Low muscle mass (SMI) | 8 |
| Lu (2024)(146) | Western Pacific Region | NO | 2017.4-2022.6 | 138 | NO | Adult | CD UC | NO | Low muscle mass (SMI), Sarcopenic obesity, Visceral obesity (VAT) | 7 |
| Mitchel (2024)(147) | Region of the Americas | YES | 2020.1-2021.12 | 1029 | NO | Children or adolescent | CD | NO | Overweight or obesity (WHO),  Malnutrition (WHO) | 8 |
| Neelam (2024)(148) | South East Asia | NO | 2021.8-2022.12 | 114 | YES | Adult | UC | YES | Sarcopenia (AWGS) | 9 |
| Oliveria (2024)(149) | Region of the Americas | NO | 2019.1-2021.12 | 140 | NO | Adult | CD UC | NO | Overweight (BMI), Obesity (BMI) | 9 |
| Park (2024)(150) | Region of the Americas | YES | 2015.10-2019.12 | 7272 | YES | Adult | CD UC | NO | Obesity (BMI) | 7 |
| Saisyo (2024)(151) | Western Pacific Region | NO | 2004.11-2021.9 | 183 | NO | Adult | CD UC | NO | Obesity (BMI) | 7 |
| Sehgal (2024)(152) | Region of the Americas | NO | 2010.1-2022.3 | 200 | NO | Adult | CD UC | NO | Overweight or obesity (BMI) | 8 |
| Sila (2024)(153) | European Region | NO | 2022.7-2023.3 | 637 | NO | Children or Adolescent | CD UC | NO | Underweight (BMI), Overweight (BMI), Obesity (BMI) | 9 |
| Sun (2024)(154) | Western Pacific Region | NO | 2012.12-2021.12 | 523 | NO | Adult | CD | NO | Underweight (BMI) | 8 |
| Umar (2024)(155) | European Region | YES | 2010.1.1-2019.12.31 | 23171 | NO | Adult | CD UC | NO | Underweight (BMI), Overweight (BMI), Obesity (BMI) | 8 |
| Zhao (2024)(156) | Western Pacific Region | NO | 2021.1-2022.3 | 212 | NO | Adult | CD | NO | Underweight (BMI), Malnutrition (GLIM) | 7 |
| Bezziom (2024)(157) | European Region | NO | 2022.12-2023.1 | 158 | NO | Adult | CD UC | NO | Underweight (BMI), Obesity (BMI), Malnutrition (GLIM) | 8 |
| Donnelly (2024)(158) | European Region | NO | 2000-2019 | 124 | NO | Adult | CD | NO | Visceral Obesity (VFA) | 7 |
| Memel (2024)(159) | Region of the Americas | NO | 2018-2023 | 290 | NO | Adult | UC | NO | Underweight (BMI), Overweight (BMI), Obesity (BMI) | 7 |
| Ndzo (2024)(160) | European Region | YES | 1942-2020 | 8797 | NO | NA | CD | NO | Underweight (BMI), Overweight (BMI), Obesity (BMI) | 8 |
| Viganò (2024)(161) | European Region | NO | 2017.1 | 295 | YES | Adult | CD UC | YES | Malnutrition (ESPEN) | 9 |
| Viser (2024)(162) | Region of the Americas | NO | 2022.6.7-2022.7.19 | 237 | NO | Adult | CD UC | NO | Underweight (BMI), Overweight (BMI), Obesity (BMI) | 8 |
| Wei (2024)(163) | Western Pacific Region | NO | 2014.11-2022.10 | 457 | YES | Adult | UC | NO | Low muscle mass (SMI), Malnutrition (GLIM) | 7 |
| Xie (2024)(164) | Western Pacific Region | NO | 2014.4-2022.12 | 117 | YES | Adult | CD | NO | Low muscle mass (SMI) | 7 |
| Yu (2024)(165) | Western Pacific Region | NO | 2022-2023 | 149 | YES | Adult | CD UC | NO | Malnutrition (GLIM) | 8 |
| Li (2024)(166) | Western Pacific Region | NO | 2016.1-2024.1 | 118 | NO | Children or Adolescent | CD UC | NO | Underweight (BMI), Overweight (BMI), Obesity (BMI) | 8 |
| Liu (2024)(167) | Western Pacific Region | NO | 2019.9-2023.3 | 115 | NO | Adult | CD UC | YES | Malnutrition (GLIM), Malnutrition (ESPEN) | 8 |
| Liu (2024)(168) | Western Pacific Region | NO | 2018.12.-2022.12 | 230 | NO | Adult | CD UC | NO | Low muscle mass (SMI) | 7 |
| Abdoh (2025)(169) | Eastern Mediterranean Region | NO | 2023.11-2024.2 | 116 | NO | Adult | CD UC | NO | Underweight (BMI), Overweight (BMI), Obesity (BMI) | 8 |
| Hsu (2025)(170) | Western Pacific Region | NO | 2015.10-2024.8 | 555 | YES | Adult | CD UC | NO | Obesity (BMI) | 7 |
| Kim (2025)(171) | Western Pacific Region | YES | 2008.1-2021.12 | 2273 | NO | Adult | CD UC | NO | Underweight (BMI), Overweight (BMI), Obesity (BMI) | 8 |
| Kochhar (2025)(172) | Region of the Americas | YES | 2015.1.1-2019.12.1 | 15177 | NO | Adult | CD UC | NO | Obesity (BMI) | 7 |
| Liu (2025)(173) | Western Pacific Region | NO | 2022.7-2023.7 | 300 | NO | Adult | CD UC | NO | Malnutrition (GLIM) | 8 |
| Mancone (2025)(174) | European Region | NO | 2022.9-2023.1 | 300 | NO | Adult | CD UC | NO | Underweight (BMI), Overweight (BMI), Obesity (BMI) | 9 |
| Naruse (2025)(175) | Western Pacific Region | NO | 2019-2021 | 104 | NO | Adult | CD UC | NO | Low muscle mass (SMI), low muscle mass with obesity (SMI+BFP) | 8 |
| Oomkens (2025)(176) | European Region | YES | NA | 1066 | NO | Adult | CD UC | NO | Overweight (BMI), Obesity (BMI) | 8 |
| Quilliot (2025)(177) | European Region | NO | 2021.11-2022.4 | 434 | NO | Adult | CD UC | NO | Malnutrition (GLIM) | 9 |
| Rudnicki (2025)(178) | Region of the Americas | NO | 2014-2021 | 346 | NO | Adult | CD UC | NO | Obesity (BMI) | 7 |
| Shen (2025)(179) | Western Pacific Region | NO | 2018.1-2022.6 | 509 | NO | Adult | CD UC | NO | Obesity (BMI) | 8 |
| Shen (2025)(180) | Western Pacific Region | NO | 2023.6-2023.12 | 160 | NO | Mixed | CD UC | NO | Malnutrition (GLIM) | 8 |
| Youn (2025)(181) | Region of the Americas | NO | 2012-2022 | 704 | YES | Adult | CD UC | NO | Underweight (BMI), Overweight (BMI), Obesity (BMI) | 8 |
| Zhang (2025)(182) | Region of the Americas | YES | ~2023.9 | 5107 | NO | Adult | CD UC | NO | Underweight (BMI), Overweight (BMI), Obesity (BMI) | 9 |
| Wang (2025)(183) | Western Pacific Region | NO | 2020.9-2024.3 | 237 | YES | Adult | CD UC | YES | Malnutrition (GLIM) | 8 |
| Yang (2025)(184) | Western Pacific Region | NO | 2022.4-2023.10 | 115 | YES | Adult | CD UC | NO | Sarcopenia (AWGS) | 8 |
| Bali (2026)(185) | Region of the Americas | NO | 2019-2021 | 124 | YES | Adult | CD UC | NO | Low muscle mass (SMI) | 7 |
| Borsato (2026)(186) | Region of the Americas | NO | 2019.1-2023.8 | 404 | YES | Adult | CD UC | NO | Obesity (BMI) | 7 |
| Desalegn (2026)(187) | African Region | NO | 2024.2-2024.7 | 242 | YES | Adult | CD UC | YES | Malnutrition (SGA) | 8 |
| Dharap (2026)(188) | South East Asia Region | NO | 2021.6.1-2022.1.30 | 117 | YES | Adult | CD UC | YES | Low muscle mass (SMI) | 8 |
| Fang (2026)(189) | Western Pacific Region | NO | 2018.12-2024.12 | 308 | NO | Adult | CD UC | NO | Low muscle mass (SMI) | 7 |
| Fiorindi (2026)(190) | European Region | NO | 2024.6-2024.8 | 705 | NO | Adult | CD UC | NO | Underweight (BMI) | 7 |
| Kaazan (2026)(191) | Western Pacific Region | YES | ~2025.5 | 7134 | YES | Adult | CD UC | NO | Underweight (BMI), Overweight (BMI), Obesity (BMI) | 7 |
| Karzan (2026)(192) | European Region | NO | 2019.12-2020.12 | 286 | NO | Adult | CD UC | NO | Malnutrition (SGA) | 9 |
| Korwel (2026)(193) | European Region | NO | 2016-2019 | 306 | NO | Adult | CD UC | NO | Malnutrition (GLIM), Underweight (BMI), Overweight (BMI), Obesity (BMI) | 9 |
| Masuda (2026)(194) | Western Pacific Region | NO | NA | 240 | NO | Adult | CD UC | NO | Malnutrition (GLIM) | 9 |
| Nigro (2026)(195) | European Region | NO | NA | 150 | NO | Adult | CD UC | NO | Sarcopenia (EWSGOP) | 8 |
| Roberts (2026)(196) | European Region | YES | NA | 6453 | YES | Adult | CD UC | NO | Obesity (BMI) | 8 |
| Roberts (2026)(196) | European Region | NO | NA | 399 | NO | Children or Adolescent | CD UC | NO | Obesity (BMI) | 8 |
| Xiang (2026)(197) | Western Pacific Region | YES | 2018.1-2022.12 | 3266 | NO | Adult | CD UC | NO | Underweight (BMI), Overweight (BMI), Obesity (BMI) | 8 |
| Yu (2026)(198) | Western Pacific Region | NO | 2023.9-2023.12 | 163 | NO | Adult | CD | NO | Malnutrition (GLIM/PG-SGA/MNN-SF) | 8 |
| Zhang (2026)(199) | European Region | YES | 2006-2010 | 4966 | NO | Adult | CD UC | NO | Underweight (BMI), Overweight (BMI), Obesity (BMI), Sarcopenia (EWSGOP) | 9 |
| Zhong (2026)(200) | Western Pacific Region | NO | 2022-2025 | 333 | YES | Adult | CD | NO | Low muscle mass (SMI) | 7 |
| Zhao (2026)(201) | Western Pacific Region | NO | 2022.3-2023.6 | 103 | NO | Adult | CD | NO | Malnutrition (GLIM) | 8 |

AWGS Asian Working Group for Sarcopenia, BMI body mass index, CD Crohn’s disease, ESPEN European Society for Clinical Nutrition and Metabolism, EWGSOP European Working Group on Sarcopenia in Older People, GLIM Global Leadership Initiative on Malnutrition, ICD International Classification of Disease, MNA mini-nutrition assessment, NA non-available, PGA-SGA Patient-Generated SGA, SGA Subjective Global Assessment, SMI skeletal muscle index, UC ulcerative colitis.

**References**

1.	Blain A, Cattan S, Beaugerie L, Carbonnel F, Gendre JP, Cosnes J. Crohn's disease clinical course and severity in obese patients. *Clinical nutrition (Edinburgh, Scotland)*. 2002 Feb;21(1):51-7.

2.	Hass DJ, Brensinger CM, Lewis JD, Lichtenstein GR. The impact of increased body mass index on the clinical course of Crohn's disease. *Clinical gastroenterology and hepatology : the official clinical practice journal of the American Gastroenterological Association*. 2006 Apr;4(4):482-8.

3.	Nguyen GC, Munsell M, Harris ML. Nationwide prevalence and prognostic significance of clinically diagnosable protein-calorie malnutrition in hospitalized inflammatory bowel disease patients. *Inflammatory bowel diseases*. 2008 Aug;14(8):1105-11.

4.	Yong-jian G, Feng Z, Jia-ming Q, Kang Y, Wei C. Screening of nutritional risk in 112 patients with inflammatory bowel disease. *Chinese Journal of Clinical Nutrition*. 2009;17(6):324-7.

5.	Steed H, Walsh S, Reynolds N. A brief report of the epidemiology of obesity in the inflammatory bowel disease population of Tayside, Scotland. *Obesity facts*. 2009;2(6):370-2.

6.	Rocha R, Santana GO, Almeida N, Lyra AC. Analysis of fat and muscle mass in patients with inflammatory bowel disease during remission and active phase. *The British journal of nutrition*. 2009 Mar;101(5):676-9.

7.	Vasseur F, Gower-Rousseau C, Vernier-Massouille G, Dupas JL, Merle V, Merlin B et al. Nutritional status and growth in pediatric Crohn's disease: a population-based study. *The American journal of gastroenterology*. 2010 Aug;105(8):1893-900.

8.	Long MD, Crandall WV, Leibowitz IH, Duffy L, del Rosario F, Kim SC et al. Prevalence and epidemiology of overweight and obesity in children with inflammatory bowel disease. *Inflammatory bowel diseases*. 2011 Oct;17(10):2162-8.

9.	Causey MW, Johnson EK, Miller S, Martin M, Maykel J, Steele SR. The impact of obesity on outcomes following major surgery for Crohn's disease: an American College of Surgeons National Surgical Quality Improvement Program assessment. *Diseases of the colon and rectum*. 2011 Dec;54(12):1488-95.

10.	Xiaohe G, Caifeng Z, Yanli Z, Yongmei Q, Yu H. Screening and analysis of nutrition risk in active ulcerative colitis. *Chin J Gastroenterol Hepatol*. 2013;22(1):71-3.

11.	Zwintscher NP, Horton JD, Steele SR. Obesity has minimal impact on clinical outcomes in children with inflammatory bowel disease. *Journal of pediatric surgery*. 2014 Feb;49(2):265-8; discussion 8.

12.	Stabroth-Akil D, Leifeld L, Pfützer R, Morgenstern J, Kruis W. The effect of body weight on the severity and clinical course of ulcerative colitis. *International journal of colorectal disease*. 2015 Feb;30(2):237-42.

13.	Seminerio JL, Koutroubakis IE, Ramos-Rivers C, Hashash JG, Dudekula A, Regueiro M et al. Impact of Obesity on the Management and Clinical Course of Patients with Inflammatory Bowel Disease. *Inflammatory bowel diseases*. 2015 Dec;21(12):2857-63.

14.	Flores A, Burstein E, Cipher DJ, Feagins LA. Obesity in Inflammatory Bowel Disease: A Marker of Less Severe Disease. *Digestive diseases and sciences*. 2015 Aug;60(8):2436-45.

15.	Manne A, Khan AS, Malik TA. Obesity and Outcome of Crohn's Associated Perianal Fistula Surgery: A Case-Control Study. *Gastroenterology research*. 2015 Dec;8(6):291-5.

16.	Dotson JL, Bricker JB, Kappelman MD, Chisolm D, Crandall WV. Assessment of Sex Differences for Treatment, Procedures, Complications, and Associated Conditions Among Adolescents Hospitalized with Crohn's Disease. *Inflammatory bowel diseases*. 2015 Nov;21(11):2619-24.

17.	Bryant RV, Ooi S, Schultz CG, Goess C, Grafton R, Hughes J et al. Low muscle mass and sarcopenia: common and predictive of osteopenia in inflammatory bowel disease. *Alimentary pharmacology & therapeutics*. 2015 May;41(9):895-906.

18.	Brown P, Clark T, Dowson G, Warren L, Hamlin J, Hull M et al. Relationship of Body Mass Index to Clinical Outcomes after Infliximab Therapy in Patients with Crohn's Disease. *Journal of Crohn's & colitis*. 2016 Oct;10(10):1144-50.

19.	Raffner Basson A, Swart R, Jordaan E, Mazinu M, Watermeyer G. Vitamin D Deficiency Increases the Risk for Moderate to Severe Disease Activity in Crohn's Disease Patients in South Africa, Measured by the Harvey Bradshaw Index. *Journal of the American College of Nutrition*. 2016;35(2):163-74.

20.	Pituch-Zdanowska A, Banaszkiewicz A, Dziekiewicz M, Łazowska-Przeorek I, Gawrońska A, Kowalska-Duplaga K et al. Overweight and obesity in children with newly diagnosed inflammatory bowel disease. *Advances in medical sciences*. 2016 Mar;61(1):28-31.

21.	Guardado J, Carchman E, Danicic AE, Salgado J, Watson AR, Celebrezze JP et al. Obesity Does Not Impact Perioperative or Postoperative Outcomes in Patients with Inflammatory Bowel Disease. *Journal of gastrointestinal surgery : official journal of the Society for Surgery of the Alimentary Tract*. 2016 Apr;20(4):725-33.

22.	Frigstad SO, Høivik M, Jahnsen J, Dahl SR, Cvancarova M, Grimstad T et al. Vitamin D deficiency in inflammatory bowel disease: prevalence and predictors in a Norwegian outpatient population. *Scandinavian journal of gastroenterology*. 2017 Jan;52(1):100-6.

23.	Casanova MJ, Chaparro M, Molina B, Merino O, Batanero R, Dueñas-Sadornil C et al. Prevalence of malnutrition and nutritional characteristics of patients with inflammatory bowel disease. *Journal of Crohn's and Colitis*. 2017;11(12):1430-9.

24.	Glassner K, Malaty HM, Abraham BP. Epidemiology and Risk Factors of Nonalcoholic Fatty Liver Disease Among Patients with Inflammatory Bowel Disease. *Inflammatory bowel diseases*. 2017 Jun;23(6):998-1003.

25.	Zhang T, Cao L, Cao T, Yang J, Gong J, Zhu W et al. Prevalence of Sarcopenia and Its Impact on Postoperative Outcome in Patients With Crohn's Disease Undergoing Bowel Resection. *J Parenter Enteral Nutr*. 2017 May;41(4):592-600.

26.	Singla MB, Eickhoff C, Betteridge J. Extraintestinal Manifestations Are Common in Obese Patients with Crohn's Disease. *Inflammatory bowel diseases*. 2017 Sep;23(9):1637-42.

27.	Pallav K, Riche D, May WL, Sanchez P, Gupta NK. Predictors of vitamin D deficiency in inflammatory bowel disease and health: A Mississippi perspective. *World journal of gastroenterology*. 2017 Jan 28;23(4):638-45.

28.	Pedersen M, Cromwell J, Nau P. Sarcopenia is a Predictor of Surgical Morbidity in Inflammatory Bowel Disease. *Inflammatory bowel diseases*. 2017 Oct;23(10):1867-72.

29.	Guerbau L, Gerard R, Duveau N, Staumont-Sallé D, Branche J, Maunoury V et al. Patients with Crohn's Disease with High Body Mass Index Present More Frequent and Rapid Loss of Response to Infliximab. *Inflammatory bowel diseases*. 2017 Oct;23(10):1853-9.

30.	Back IR, Marcon SS, Gaino NM, Vulcano DSB, Dorna MS, Sassaki LY. BODY COMPOSITION IN PATIENTS WITH CROHN'S DISEASE AND ULCERATIVE COLITIS. *Arquivos de gastroenterologia*. 2017 Apr-Jun;54(2):109-14.

31.	Bryant RV, Schultz CG, Ooi S, Goess C, Costello SP, Vincent AD et al. Obesity in Inflammatory Bowel Disease: Gains in Adiposity despite High Prevalence of Myopenia and Osteopenia. *Nutrients*. 2018 Sep 1;10(9).

32.	Chandradas S, Khalili H, Ananthakrishnan A, Wayman C, Reidel W, Waalen J et al. Does Obesity Influence the Risk of Clostridium difficile Infection Among Patients with Ulcerative Colitis? *Digestive diseases and sciences*. 2018 Sep;63(9):2445-50.

33.	Jain A, Bricker J, Kappelman MD, Dotson JL. Obesity Does Not Affect Early Outcomes in Children With Newly Diagnosed Crohn Disease. *Journal of pediatric gastroenterology and nutrition*. 2018 Nov;67(5):622-5.

34.	Ladd MR, Garcia AV, Leeds IL, Haney C, Oliva-Hemker MM, Alaish S et al. Malnutrition increases the risk of 30-day complications after surgery in pediatric patients with Crohn disease. *Journal of pediatric surgery*. 2018 Nov;53(11):2336-45.

35.	Madsen KG, Pottegård A, Hallas J, Kjeldsen J. Treatment Failure of TNF-α Inhibitors in Obese Patients With Inflammatory Bowel Disease-A Cohort Study. *Inflammatory bowel diseases*. 2018 Nov 29;24(12):2628-33.

36.	Spooren C, Wintjens DSJ, de Jong MJ, van der Meulen-de Jong AE, Romberg-Camps MJ, Becx MC et al. Risk of impaired nutritional status and flare occurrence in IBD outpatients. *Digestive and liver disease : official journal of the Italian Society of Gastroenterology and the Italian Association for the Study of the Liver*. 2019 Sep;51(9):1265-9.

37.	Marcil V, Levy E, Amre D, Bitton A, De Araújo Sant'Anna AMG, Szilagy A et al. A cross-sectional study on malnutrition in inflammatory bowel disease: Is there a difference based on pediatric or adult age grouping? *Inflammatory bowel diseases*. 2019;25(8):1428-41.

38.	Lomer MCE, Cahill O, Baschali A, Partha Sarathy P, Sarantidou M, Mantzaris GJ et al. A multicentre Study of Nutrition Risk Assessment in Adult Patients with Inflammatory Bowel Disease Attending Outpatient Clinics. *Annals of nutrition & metabolism*. 2019;74(1):18-23.

39.	Magrì S, Paduano D, Chicco F, Cingolani A, Farris C, Delogu G et al. Nonalcoholic fatty liver disease in patients with inflammatory bowel disease: Beyond the natural history. *World journal of gastroenterology*. 2019 Oct 7;25(37):5676-86.

40.	Yuqi Q, Mi Z, Jun S, Zhihua R. Bioelectrical Impedance Analysis for Body Composition and Nutritional Status in Hospitalized Patients With Inflammatory Bowel Disease. *Chin J Gastroenterol*. 2019;24(1):5-9.

41.	Nguyen NH, Ohno-Machado L, Sandborn WJ, Singh S. Obesity Is Independently Associated With Higher Annual Burden and Costs of Hospitalization in Patients With Inflammatory Bowel Diseases. *Clinical gastroenterology and hepatology : the official clinical practice journal of the American Gastroenterological Association*. 2019 Mar;17(4):709-18.e7.

42.	Mentella MC, Scaldaferri F, Pizzoferrato M, Gasbarrini A, Miggiano GAD. The Association of Disease Activity, BMI and Phase Angle with Vitamin D Deficiency in Patients with IBD. *Nutrients*. 2019 Oct 26;11(11).

43.	McKenna NP, Habermann EB, Zielinski MD, Lightner AL, Mathis KL. Body mass index: Implications on disease severity and postoperative complications in patients with Crohn's disease undergoing abdominal surgery. *Surgery*. 2019 Oct;166(4):703-8.

44.	Jain A, Nguyen NH, Proudfoot JA, Martin CF, Sandborn WJ, Kappelman MD et al. Impact of Obesity on Disease Activity and Patient-Reported Outcomes Measurement Information System (PROMIS) in Inflammatory Bowel Diseases. *The American journal of gastroenterology*. 2019 Apr;114(4):630-9.

45.	Pizzoferrato M, de Sire R, Ingravalle F, Mentella MC, Petito V, Martone AM et al. Characterization of Sarcopenia in an IBD Population Attending an Italian Gastroenterology Tertiary Center. *Nutrients*. 2019 Sep 24;11(10).

46.	Shrestha MP, Taleban S. Obesity Is Associated with Increased Risk of Colectomy in Inflammatory Bowel Disease Patients Hospitalized with Clostridium difficile Infection. *Digestive diseases and sciences*. 2019 Jun;64(6):1632-9.

47.	Chandrakumar A, Wang A, Grover K, El-Matary W. Obesity Is More Common in Children Newly Diagnosed With Ulcerative Colitis as Compared to Those With Crohn Disease. *Journal of pediatric gastroenterology and nutrition*. 2020 May;70(5):593-7.

48.	Jinjie S, Yongqiang S. Investigation on nutritional cognitive behavior of patients with inflammatory bowel disease and malnutrition analysis. *Journal of Clinical Medicine in Practice*. 2020;24(2):83-6.

49.	Kang MK, Kim KO, Kim MC, Park JG, Jang BI. Sarcopenia Is a New Risk Factor of Nonalcoholic Fatty Liver Disease in Patients with Inflammatory Bowel Disease. *Digestive diseases (Basel, Switzerland)*. 2020;38(6):507-14.

50.	Xing Z, Xianghong Y, Jinzhong X, Yun L, Xianyu W, Lin S. Sarcopenia and its determinants in patients with Crohn's disease. *Journal of Nursing Science*. 2020;35(13 ):19-23.

51.	Labarthe G, Dolores M, Verdalle-Cazes M, Charpentier C, Roullee P, Dacher JN et al. Magnetic resonance imaging assessment of body composition parameters in Crohn's disease. *Digestive and liver disease : official journal of the Italian Society of Gastroenterology and the Italian Association for the Study of the Liver*. 2020 Aug;52(8):878-84.

52.	Greuter T, Porchet F, Braga-Neto MB, Rossel JB, Biedermann L, Schreiner P et al. Impact of obesity on disease activity and disease outcome in inflammatory bowel disease: Results from the Swiss inflammatory bowel disease cohort. *United European gastroenterology journal*. 2020 Dec;8(10):1196-207.

53.	El Mouzan M, Alahmadi N, Alsaleeem KA, Assiri A, AlSaleem B, Al Sarkhy A. Prevalence of nutritional disorders in Saudi children with inflammatory bowel disease based on the national growth reference. *Arab Journal of Gastroenterology*. 2020;21(3):179-82.

54.	Cioffi I, Marra M, Imperatore N, Pagano MC, Santarpia L, Alfonsi L et al. Assessment of bioelectrical phase angle as a predictor of nutritional status in patients with Crohn's disease: A cross sectional study. *Clinical nutrition (Edinburgh, Scotland)*. 2020 May;39(5):1564-71.

55.	Lin S, Xing Z, Jin-zhong X, Ru-ting S, Xiang-hong Y. Factors related to fatigue in patients with Crohn's disease. *Parenteral & Enteral Nutrition*. 2020;27(3):176-80.

56.	Pulley J, Todd A, Flatley C, Begun J. Malnutrition and quality of life among adult inflammatory bowel disease patients. *JGH open : an open access journal of gastroenterology and hepatology*. 2020 Jun;4(3):454-60.

57.	Yerushalmy-Feler A, Galai T, Moran-Lev H, Ben-Tov A, Dali-Levy M, Weintraub Y et al. BMI in the lower and upper quartiles at diagnosis and at 1-year follow-up is significantly associated with higher risk of disease exacerbation in pediatric inflammatory bowel disease. *European journal of pediatrics*. 2021 Jan;180(1):21-9.

58.	Ünal NG, Oruç N, Tomey O, Özütemiz AÖ. Malnutrition and sarcopenia are prevalent among inflammatory bowel disease patients with clinical remission. *European journal of gastroenterology & hepatology*. 2021;33(11):1367-75.

59.	Prieto JMI, Andrade AR, Magro DO, Imbrizi M, Nishitokukado I, Ortiz-Agostinho CL et al. Nutritional Global Status and Its Impact in Crohn's Disease. *Journal of the Canadian Association of Gastroenterology*. 2021;4(6):290-5.

60.	Johnson AM, Harmsen WS, Aniwan S, Tremaine WJ, Abu Dayyeh BK, Loftus EV. Prevalence and Impact of Obesity on Disease-specific Outcomes in a Population-based Cohort of Patients with Ulcerative Colitis. *Journal of Crohn's & colitis*. 2021 Nov 8;15(11):1816-23.

61.	Kim SK, Lee HS, Kim BJ, Park JH, Hwang SW, Yang DH et al. The Clinical Features of Inflammatory Bowel Disease in Patients with Obesity. *Canadian journal of gastroenterology & hepatology*. 2021;2021:9981482.

62.	Bian D, Jiang Y, Gu Y, He Z, Chen Q, Tang Y et al. Body Mass Index and Disease Activity Are Associated With Moderate to Severe Disability in Crohn's Disease: A Cross-Sectional Study in Shanghai. *Frontiers in medicine*. 2021;8:662488.

63.	Weissman S, Patel K, Kolli S, Lipcsey M, Qureshi N, Elias S et al. Obesity in Inflammatory Bowel Disease Is Associated with Early Readmissions Characterised by an Increased Systems and Patient-level Burden. *Journal of Crohn's & colitis*. 2021 Nov 8;15(11):1807-15.

64.	Wan Y, Zhang D, Xing T, Liu Q, Chi Y, Zhang H et al. The impact of visceral obesity on chronic constipation, inflammation, immune function and cognitive function in patients with inflammatory bowel disease. *Aging*. 2021 Feb 17;13(5):6702-11.

65.	Singh S, Heien HC, Sangaralingham L, Shah ND, Sandborn WJ. Obesity Is Not Associated With an Increased Risk of Serious Infections in Biologic-Treated Patients With Inflammatory Bowel Diseases. *Clinical and translational gastroenterology*. 2021 Jul 6;12(7):e00380.

66.	Taylor LM, Eslamparast T, Farhat K, Kroeker K, Halloran B, Shommu N et al. Using Patient Completed Screening Tools to Predict Risk of Malnutrition in Patients With Inflammatory Bowel Disease. *Crohn's & colitis 360*. 2021 Jul;3(3):otab043.

67.	Marino LV, Ashton JJ, Beattie RM. The impact of national lockdown on nutritional status of children with inflammatory bowel disease. *Journal of human nutrition and dietetics : the official journal of the British Dietetic Association*. 2021 Aug;34(4):656-9.

68.	Park YE, Park SJ, Park JJ, Cheon JH, Kim TI, Kim WH. Incidence and risk factors of micronutrient deficiency in patients with IBD and intestinal Behçet’s disease: folate, vitamin B12, 25-OH-vitamin D, and ferritin. *BMC gastroenterology*. 2021;21(1).

69.	El-Dallal M, Stein DJ, Raita Y, Feuerstein JD. The impact of obesity on hospitalized patients with ulcerative colitis. *Annals of gastroenterology*. 2021;34(2):196-201.

70.	Egberg MD, Galanko JA, Banegas M, Roberson M, Strassle PD, Phillips M et al. Obesity Increases the Risk of Hospital Readmission Following Intestinal Surgery for Children With Crohn Disease. *Journal of pediatric gastroenterology and nutrition*. 2021 Nov 1;73(5):620-5.

71.	Vohra I, Attar B, Haghbin H, Mutneja H, Katiyar V, Sharma S et al. Incidence and risk factors for 30-day readmission in ulcerative colitis: nationwide analysis in biologic era. *European journal of gastroenterology & hepatology*. 2021 Sep 1;33(9):1174-84.

72.	Zhang C, Yu D, Hong L, Zhang T, Liu H, Fan R et al. Prevalence of Sarcopenia and Its Effect on Postoperative Complications in Patients with Crohn's Disease. *Gastroenterology research and practice*. 2021;2021:3267201.

73.	Chuck W, Shadbolt BF, Nordin F, Subramaniam K. BMI is important in predicting the loss of response in inflammatory bowel disease patients on tumour necrosis factor- inhibitors. *European journal of gastroenterology & hepatology*. 2022;34(6):622-9.

74.	Shen X, Liu S, Chen X, Geng B, Tian Z, Ding X. Application of Global Leadership Initiative on Malnutrition criteria for the diagnosis of malnutrition in patients with ulcerative colitis. *Chinese Journal of Clinical Nutrition*. 2022;30(5):266-72.

75.	Trinder MW, Clifford M, Jones AL, Shepherd T, Jacob AO. The impact of sarcopenia on outcomes in patients with inflammatory bowel disease undergoing colorectal surgery. *ANZ journal of surgery*. 2022 Mar;92(3):397-402.

76.	Liu J, Ge X, Ouyang C, Wang D, Zhang X, Liang J et al. Prevalence of Malnutrition, Its Risk Factors, and the Use of Nutrition Support in Patients with Inflammatory Bowel Disease. *Inflammatory bowel diseases*. 2022 Jun 2;28(Suppl 2):S59-s66.

77.	Johnson AM, Harmsen WS, Aniwan S, Tremaine WJ, Raffals LE, Abu Dayyeh BK et al. Prevalence and Impact of Obesity in a Population-Based Cohort of Patients With Crohn's Disease. *Journal of clinical gastroenterology*. 2022 Dec 30.

78.	Gordon BL, Galati JS, Yang S, Longman RS, Lukin D, Scherl EJ et al. Prevalence and factors associated with vitamin C deficiency in inflammatory bowel disease. *World journal of gastroenterology*. 2022 Sep 7;28(33):4834-45.

79.	Abd-El-aziz MA, Hübner M, Demartines N, Larson DW, Grass F. Simple Clinical Screening Underestimates Malnutrition in Surgical Patients with Inflammatory Bowel Disease—An ACS NSQIP Analysis. *Nutrients*. 2022;14(5).

80.	Aljohani RS, Alaklabi A, Alsitary YM, bin Khunayn MA, Hijazi SO, Alshagary RI et al. Clinical profile, course and outcomes of adults with inflammatory bowel disease over a decade: a single center experience. *Annals of Saudi medicine*. 2022;42(6):397-407.

81.	Yelencich E, Truong E, Widaman AM, Pignotti G, Yang L, Jeon Y et al. Avoidant Restrictive Food Intake Disorder Prevalent Among Patients With Inflammatory Bowel Disease. *Clinical gastroenterology and hepatology : the official clinical practice journal of the American Gastroenterological Association*. 2022 Jun;20(6):1282-9.e1.

82.	Zhang Y, Zhang L, Gao X, Dai C, Huang Y, Wu Y et al. Validation of the GLIM criteria for diagnosis of malnutrition and quality of life in patients with inflammatory bowel disease: A multicenter, prospective, observational study. *Clinical nutrition (Edinburgh, Scotland)*. 2022 Jun;41(6):1297-306.

83.	Xu F, Hu J, Yang Q, Ji Y, Cheng C, Zhu L et al. Prevalence and factors associated with fatigue in patients with ulcerative colitis in China: a cross-sectional study. *BMC gastroenterology*. 2022 Jun 3;22(1):281.

84.	von Graffenried T, Schoepfer AM, Rossel JB, Greuter T, Safroneeva E, Godat S et al. Impact of Overweight and Obesity on Disease Outcome in the Pediatric Swiss Inflammatory Bowel Disease Cohort. *JPGN reports*. 2022 May;3(2):e193.

85.	Tseng Y, Mo S, Zeng Y, Zheng W, Song H, Zhong B et al. Machine Learning Model in Predicting Sarcopenia in Crohn's Disease Based on Simple Clinical and Anthropometric Measures. *International journal of environmental research and public health*. 2022 Dec 30;20(1).

86.	Peng Z, Xu D, Li Y, Peng Y, Liu X. Phase Angle as a Comprehensive Tool for Nutritional Monitoring and Management in Patients with Crohn's Disease. *Nutrients*. 2022 May 28;14(11).

87.	Reiner J, Koch K, Woitalla J, Huth A, Bannert K, Sautter L et al. Body impedance analysis to estimate malnutrition in inflammatory bowel disease patients – A cross-sectional study. *Journal of digestive diseases*. 2022;23(12):687-94.

88.	Liu S, Ding X, Maggiore G, Pietrobattista A, Satapathy SK, Tian Z et al. Sarcopenia is associated with poor clinical outcomes in patients with inflammatory bowel disease: a prospective cohort study. *Annals of translational medicine*. 2022 Mar;10(6):367.

89.	Ohya H, Kimura H, Watanabe J, Nakagawa K, Suwa Y, Ozawa M et al. Preoperative prevalence and risk factors of deep-vein thrombosis in Japanese surgical patients with ulcerative colitis: a retrospective investigational study. *Surgery today*. 2022 Feb;52(2):251-9.

90.	Kuloglu Z, Çetin F, Urgancı N, Önal Z, Sarı S, Yüksekkaya H et al. Nutritional characteristic of children with inflammatory bowel disease in the nationwide inflammatory bowel disease registry from the Mediterranean region. *European journal of clinical nutrition*. 2022;76(9):1289-96.

91.	Li Y, Peng Z, Xu D, Peng Y, Liu X. The GLIM Criteria Represent a More Appropriate Tool for Nutritional Assessment in Patients With Crohn's Disease. *Frontiers in nutrition*. 2022;9:826028.

92.	Karachaliou A, Yannakoulia M, Bletsa M, Mantzaris GJ, Archavlis E, Karampekos G et al. Assessment of Dietary Adequacy and Quality in a Sample of Patients with Crohn's Disease. *Nutrients*. 2022 Dec 9;14(24).

93.	Kadenczki O, Dezsofi A, Cseh A, Szucs D, Vass N, Nemes E et al. Disease Activity Is Associated with Obesity in Newly Diagnosed Pediatric Patients with Ulcerative Colitis. *International journal of environmental research and public health*. 2022 Dec 1;19(23).

94.	Jin W, Yang DH, Tchah H, Kwon KA, Kim JH, Jeong SJ et al. Wasting condition as a marker for severe disease in pediatric Crohn's disease. *Medicine (United States)*. 2022;101(20).

95.	Hoffman K, Mansoor E, Panhwar MS, Regueiro M, Cooper G, Qazi T. Prevalence of Obstructive Sleep Apnea Is Increased in Patients With Inflammatory Bowel Disease: A Large, Multi-Network Study. *Crohn's & colitis 360*. 2022 Jul;4(3):otac026.

96.	Gu P, Luo J, Kim J, Paul P, Limketkai B, Sauk JS et al. Effect of Obesity on Risk of Hospitalization, Surgery, and Serious Infection in Biologic-Treated Patients With Inflammatory Bowel Diseases: A CA-IBD Cohort Study. *The American journal of gastroenterology*. 2022 Oct 1;117(10):1639-47.

97.	Jain A, Bricker J, Kappelman MD, Dotson JL. Overweight and Obese Status Is Not Associated With Disease Activity for Children and Adolescents With Newly Diagnosed Inflammatory Bowel Disease. *The American journal of gastroenterology*. 2022 Jul 1;117(7):1146-53.

98.	Ge X, Xia J, Wu Y, Ye L, Liu W, Qi W et al. Sarcopenia assessed by computed tomography is associated with colectomy in patients with acute severe ulcerative colitis. *European journal of clinical nutrition*. 2022 Mar;76(3):410-8.

99.	Dahiya DS, Kichloo A, Wani F, Singh J, Solanki D, Shaka H. A nationwide analysis on the influence of obesity in inflammatory bowel disease hospitalizations. *Intestinal research*. 2022 Jul;20(3):342-9.

100.	Campbell JP, Teigen L, Manski S, Blumhof B, Guglielmo FF, Shivashankar R et al. Sarcopenia Is More Prevalent Among Inflammatory Bowel Disease Patients Undergoing Surgery and Predicts Progression to Surgery Among Medically Treated Patients. *Inflammatory bowel diseases*. 2022 Dec 1;28(12):1844-50.

101.	Shehab M, Alali A, Al-Hindawi A, Alsayegh A, Aldallal U, Abdullah I et al. Impact of tumor necrosis factor antagonist combination and anti-integrin therapies on body mass index in inflammatory bowel disease: A cross-sectional study. *Frontiers in medicine*. 2022;9:1045661.

102.	Wang M, Guo Q, Liu H, Liu M, Tang C, Wu J et al. GLIM criteria using NRS-2002 and MUST as the first step adequately diagnose the malnutrition in Crohn's disease inpatients: A retrospective study. *Frontiers in nutrition*. 2022;9:1059191.

103.	Abu-Freha N, Ealiwa N, AbuTailakh M, Abu-Abed M, Bader S, Tabu R et al. Ethnic Issues and Disparities in Inflammatory Bowel Diseases: What Can We Learn from the Arab Population in Israel? *Journal of personalized medicine*. 2023 Jun 17;13(6).

104.	Wei W, Yan P, Wang F, Bai X, Wang J, Li J et al. Malnutrition Defined by the Global Leadership Initiative on Malnutrition (GLIM) Criteria in Hospitalized Patients with Ulcerative Colitis and Its Association with Clinical Outcomes. *Nutrients*. 2023 Aug 14;15(16).

105.	Wang G, Yang J, Xu C, Zhang X, Zhou H, Zhang M. Analysis of malnutrition as per global leadership initiative on malnutrition criteria and its influencing factors in inflammatory bowel disease patients from a tertiary hospital in Shenzhen. *Chinese Journal of Clinical Nutrition*. 2023;31(1):9-17.

106.	Singh A, Midha V, Mahajan R, Verma S, Kakkar C, Grover J et al. Evaluation of Nutritional Characteristics Reveals Similar Prevalence of Malnutrition in Patients with Ulcerative Colitis and Crohn's Disease. *Digestive diseases and sciences*. 2023 Feb;68(2):580-95.

107.	Tang W, Xie G, Li J, Tan W, Yi R, Yang L et al. Body composition parameters correlate with the endoscopic severity in Crohn's disease patients treated with infliximab. *Frontiers in nutrition*. 2023;10:1251448.

108.	Sim B, Cheah MCC, Chan WPW, Wang YT, Tan MTK, Shim HH et al. Approximately 1 in 10 ambulatory inflammatory bowel disease patients are at risk of malnutrition: A prospective Singapore cohort. *Journal of digestive diseases*. 2023 Jan;24(1):10-8.

109.	Sehgal P, Shen B, Li J, Freedberg DE. Obesity among those newly diagnosed with Crohn's disease and ulcerative colitis compared with the general population. *Frontline gastroenterology*. 2023;14(4):319-25.

110.	Gold SL, Rabinowitz LG, Manning L, Keefer L, Rivera-Carrero W, Stanley S et al. High Prevalence of Malnutrition and Micronutrient Deficiencies in Patients With Inflammatory Bowel Disease Early in Disease Course. *Inflammatory bowel diseases*. 2023 Mar 1;29(3):423-9.

111.	Elangovan A, Shah R, Ali SMJ, Katz J, Cooper GS. High Burden of Obesity and Low Rates of Weight Loss Pharmacotherapy in Inflammatory Bowel Disease: 10-Year Trend. *Crohn's & colitis 360*. 2023 Apr;5(2):otad007.

112.	Fernandez C, Gajic Z, Esen E, Remzi F, Hudesman D, Adhikari S et al. Preoperative Risk Factors for Adverse Events in Adults Undergoing Bowel Resection for Inflammatory Bowel Disease: 15-Year Assessment of the American College of Surgeons National Surgical Quality Improvement Program. *The American journal of gastroenterology*. 2023 Dec 1;118(12):2230-41.

113.	Dua A, Corson M, Sauk JS, Jaffe N, Limketkai BN. Impact of malnutrition and nutrition support in hospitalised patients with inflammatory bowel disease. *Alimentary pharmacology & therapeutics*. 2023 Apr;57(8):897-906.

114.	Bacha RA, Bouhnik Y, Serrero M, Filippi J, Roblin X, Bourrier A et al. Obesity in adult patients with inflammatory bowel disease: Clinical features and impact on disability. A cross-sectional survey from the GETAID. *Digestive and liver disease : official journal of the Italian Society of Gastroenterology and the Italian Association for the Study of the Liver*. 2023 May 26.

115.	Jun Z. Nutritional Risk Screening and Risk Factor Analysis in Active Ulcerative Colitis. *Modern Diagnosis and Treatment*. 2023;34(2):251-3(In Chinese).

116.	Wang Y, Yao D, He Y, He Q, Li Y. Earlier anti-TNF therapy reduces the risk of malnutrition associated with alterations in body composition in patients with Crohn's disease. *Frontiers in nutrition*. 2023;10:1114758.

117.	Wang Y, Liu L, Han J, Fan X, Guo Q, Wu Z. Evaluation the Impact of Obesity and Different Metabolic Status on the Prognosis of Hospitalized Patients with Inflammatory Bowel Disease: A Cohort Study. *Obesity facts*. 2023 Aug 8.

118.	Nam K, Lee JY, Ko Y, Kim KW, Lee HS, Hong SW et al. Impact of Sarcopenia on Clinical Course of Inflammatory Bowel Disease in Korea. *Digestive diseases and sciences*. 2023 Jun;68(6):2165-79.

119.	Grova M, Crispino F, Maida M, Vitello A, Renna S, Casà A et al. Sarcopenia is a negative predictive factor for endoscopic remission in patients with Crohn's disease treated with biologics. *Digestive and liver disease : official journal of the Italian Society of Gastroenterology and the Italian Association for the Study of the Liver*. 2023 Jul;55(7):865-71.

120.	Cankurtaran RE, Güneş YC, Dirican E, Algın O, Cankurtaran D, Tayfur Yürekli Ö. Sarcopenia and Myosteatosis Assessed by Magnetic Resonance Enterography May Predict Negative Outcomes in Patients with Crohn's Disease. *The Turkish journal of gastroenterology : the official journal of Turkish Society of Gastroenterology*. 2023 Aug;34(8):839-49.

121.	Barajas Ordonez F, Melekh B, Rodríguez-Feria P, Melekh O, Thormann M, Damm R et al. Body Composition Predictors of Complicated Crohn's Disease. *Digestive diseases (Basel, Switzerland)*. 2023;41(4):589-99.

122.	Barnes A, Andrews JM, Mukherjee S, Bryant RV, Bampton P, Spizzo P et al. Simple Novel Screening Tool for Obstructive Sleep Apnea in Inflammatory Bowel Disease. *Crohn's & colitis 360*. 2023 Apr;5(2):otad016.

123.	Bedard K, Taylor L, Rajabali N, Kroeker K, Halloran B, Meng G et al. Handgrip strength and risk of malnutrition are associated with an increased risk of hospitalizations in inflammatory bowel disease patients. *Therapeutic advances in gastroenterology*. 2023;16:17562848231194395.

124.	Choi EJ, Baek DH, Lee HS, Song GA, Kim TO, Park YE et al. The effect of biological agent on body composition in patients with Crohn's disease. *BMC gastroenterology*. 2023 Mar 30;23(1):100.

125.	Cioffi I, Scialò F, Di Vincenzo O, Gelzo M, Marra M, Testa A et al. Serum Interleukin 6, Controlling Nutritional Status (CONUT) Score and Phase Angle in Patients with Crohn's Disease. *Nutrients*. 2023 Apr 18;15(8).

126.	García-Mateo S, Martínez-Domínguez SJ, Gargallo-Puyuelo CJ, Arroyo Villarino MT, Laredo De La Torre V, Gallego B et al. Lifestyle Can Exert a Significant Impact on the Development of Metabolic Complications and Quality Life in Patients with Inflammatory Bowel Disease. *Nutrients*. 2023 Sep 14;15(18).

127.	Huang L, Niannian F, Zhang Y, Shi Y, Gao X, Zhang L et al. Rapid assessment of malnutrition based on GLIM diagnosis in Crohn's disease. *Frontiers in nutrition*. 2023;10:1236036.

128.	Kim M, Cho M, Hong S, Song JH, Kim ER, Hong SN et al. Weight loss from diagnosis of Crohn's disease to one year post-diagnosis results in earlier surgery. *Scientific reports*. 2023 Nov 30;13(1):21101.

129.	Lee JM, Han K, Lee KM, Yun JS. Fracture Risk in Middle-Aged and Older Patients With Inflammatory Bowel Disease: A Korean Nationwide Population-Based Cohort Study. *Journal of Korean medical science*. 2023 Sep 4;38(35):e275.

130.	Lima JS, de Brito CAA, Celani LMS, de Araújo MVT, de Lucena MT, Vasconcelos GBS et al. Body Mass Index Profile of Adult Patients with Inflammatory Bowel Disease in a Multicenter Study in Northeastern Brazil. *Clinical and experimental gastroenterology*. 2023;16:213-24.

131.	Traiki TAB, Alshammari SA, Aljomah NA, Alsalouli MM, Altawil ES, Abdulla MH et al. The impact of preoperative total parenteral nutrition on the surgical complications of Crohn's disease: A retrospective cohort study. *Saudi journal of gastroenterology : official journal of the Saudi Gastroenterology Association*. 2023 May-Jun;29(3):158-63.

132.	Wall CL, Wilson B, Lomer MCE. Development and validation of an inflammatory bowel disease nutrition self-screening tool (IBD-NST) for digital use. *Frontiers in nutrition*. 2023;10:1065592.

133.	Yoon H, Park YS, Shin JE, Ye BD, Eun CS, Yoon SM et al. Clinical importance of weight gain and associated factors in patients with moderate to severe ulcerative colitis: results from the MOSAIK cohort in Korea. *BMC gastroenterology*. 2023 Nov 21;23(1):405.

134.	Zhang Y, Zhang L, Gao X, Dai C, Huang Y, Wu Y et al. Impact of malnutrition and sarcopenia on quality of life in patients with inflammatory bowel disease: A multicentre study. *Journal of cachexia, sarcopenia and muscle*. 2023 Dec;14(6):2663-75.

135.	Zhi J, Jiāo B, Qing S, Liang L. Factors associated with low skeletal muscle index among patients with Crohn's disease. *Revista da Associacao Medica Brasileira (1992)*. 2023;69(7):e20221606.

136.	Zhou J, Xiao X, Xia Y, You J, Zhao H. NUTRITIONAL STATUS AND INFLUENCING FACTORS IN CHILDREN WITH NEWLY DIAGNOSED INFLAMMATORY BOWEL DISEASE. *Journal of pediatric gastroenterology and nutrition*. 2023;76:1195.

137.	Yuting S, Pei Z, Dongting G, Weidong S. Analysis of simple diagnostic indexes of Crohn's disease complicated with sarcopenia. *Chinese Journal of Inflammatory Bowel Diseases*. 2023;7(2):151-6.

138.	Pei Z, Weidong S, Xia L, Yuting S, Xinpu Y. Study on risk factors for sarcopenia in Crohn's disease. *Journal of Southeast University(Medical Science Edition)*. 2023;42(6):896-902.

139.	Algin O, Güneş YC, Cankurtaran RE, Corabay S, Yurekli OT. The Relationship Between Intramural Fat Accumulation and Sarcopenia on MR Enterography Exams in Patients with Crohn's Disease. *RoFo : Fortschritte auf dem Gebiete der Rontgenstrahlen und der Nuklearmedizin*. 2024 Jul 8.

140.	Desai A, Petrov J, Hashash JG, Patel H, Brahmbhatt B, Kochhar GS et al. Use of glucagon-like peptide-1 receptor agonists for type 2 diabetes mellitus and outcomes of inflammatory bowel disease. *Alimentary pharmacology & therapeutics*. 2024 Sep;60(5):620-32.

141.	Ebach DR, Jester TW, Galanko JA, Firestine AM, Ammoury R, Cabrera J et al. High Body Mass Index and Response to Anti-Tumor Necrosis Factor Therapy in Pediatric Crohn's Disease. *American Journal of Gastroenterology*. 2024;119(6):1110-6.

142.	Ergenc I, Ismail Basa C, Uzum A, Sahin S, Kani HT, Aslan R et al. High prevalence of muscle weakness and probable sarcopenia in patients with inflammatory bowel disease. *Nutrition in clinical practice : official publication of the American Society for Parenteral and Enteral Nutrition*. 2024 Jun;39(3):557-67.

143.	Hu N, Liu J, Gao X, Tang H, Wang J, Wei Z et al. The association between hemoglobin level and sarcopenia in Chinese patients with Crohn’s disease. *BMC gastroenterology*. 2024;24(1).

144.	Kodia K, Huerta CT, Alnajar A, Collins S, Ribieras A, Horner LP et al. Outcomes Among Malnourished Patients With Crohn's Disease Undergoing Elective Ileocecectomy: A Nationwide Analysis. *The American surgeon*. 2024 Apr;90(4):739-47.

145.	Li S, Wu H, Miao S, Huang C, Zhang Y, Shao X et al. CT-based body composition parameters predict the loss of response to infliximab in patients with Crohn's disease. *The American journal of the medical sciences*. 2024 Sep 3.

146.	Lu J, Xu H, Shi H, Zheng J, Cheng T, Zhou M et al. Computed tomography-based body composition parameters can predict short-term prognosis in ulcerative colitis patients. *Insights into imaging*. 2024 Feb 27;15(1):60.

147.	Mitchel EB, Huang J, Zemel B, Baldassano R, Albenberg L, Denburg M. Trajectory of body mass index and obesity in children with Crohn's disease compared to healthy children. *Journal of pediatric gastroenterology and nutrition*. 2024 Jan;78(1):85-94.

148.	Neelam PB, Pal R, Gupta P, Singh AK, Shah J, Mandavdhare HS et al. Sarcopenia is common in ulcerative colitis and correlates with disease activity. *Intestinal research*. 2024 Apr;22(2):162-71.

149.	Oliveira L, Ribeiro T, Mourao Junior CA, Barra MAL, Silva MHG, Shafee LP et al. Metabolic dysfunction-associated steatotic liver disease prevalence and risk factors in inﬂammatory bowel disease in tertiary center. *Revista da Associacao Medica Brasileira (1992)*. 2024;70(6):e20231321.

150.	Park L, McKechnie T, Lee Y, Tessier L, Passos E, Doumouras A et al. Short-term postoperative outcomes for obese versus non-obese inflammatory bowel disease patients undergoing bowel resection: a propensity score matched analysis. *International journal of colorectal disease*. 2024;39(1).

151.	Saisyo A, Hashimoto S, Ishida H, Kashibe K, Oka T, Hirano Y et al. Treatment of Inflammatory Bowel Disease with Biologics in Japan: A Single-Center, Retrospective Pharmacoeconomic Study. *Biological & pharmaceutical bulletin*. 2024;47(1):232-9.

152.	Sehgal P, Su S, Zech J, Nobel Y, Luk L, Economou I et al. Visceral Adiposity Independently Predicts Time to Flare in Inflammatory Bowel Disease but Body Mass Index Does Not. *Inflammatory bowel diseases*. 2024 Apr 3;30(4):594-601.

153.	Sila S, Aloi M, Cucinotta U, Gianolio L, Granot M, Hradsky O et al. Effect of Overweight and Obesity on the Response to Anti-TNF Therapy and Disease Course in Children With IBD. *Inflammatory bowel diseases*. 2024 Jul 31.

154.	Sun Z, Cao L, Chen Y, Zhu W, Li Y. Long-term outcomes of intestinal penetrating Crohn's disease following successful nonoperative management. *European journal of gastroenterology & hepatology*. 2024;36(7):867-74.

155.	Umar N, Harvey P, Adderley NJ, Haroon S, Trudgill N. The Time to Inflammatory Bowel Disease Diagnosis for Patients Presenting with Abdominal Symptoms in Primary Care and its Association with Emergency Hospital Admissions and Surgery: A Retrospective Cohort Study. *Inflammatory bowel diseases*. 2024 Apr 2.

156.	Ze-hua Z, Chu-lin C, Xiang-hong Y, Yi L. Comparison of the performance of different nutritional screening tools for preoperative patients with Crohn's disease. *Parenteral & Enteral Nutrition*. 2024;31(2):114-7,23.

157.	Bezzio C, Brinch D, Ribaldone DG, Cappello M, Ruzzon N, Vernero M et al. Prevalence, Risk Factors and Association with Clinical Outcomes of Malnutrition and Sarcopenia in Inflammatory Bowel Disease: A Prospective Study. *Nutrients*. 2024 Nov 21;16(23).

158.	Donnelly M, Driever D, Ryan É J, Elliott JA, Finnegan J, McNamara D et al. Obesity, Sarcopenia and Myosteatosis: Impact on Clinical Outcomes in the Operative Management of Crohn's Disease. *Inflammatory bowel diseases*. 2024 Sep 3;30(9):1517-28.

159.	Memel Z, Thiemann A, Dort C, Mahadevan U, Beck KR. Prevalence of Malnutrition and Micronutrient Deficiencies in Older Adults with Ulcerative Colitis. *Digestive diseases and sciences*. 2024 Nov;69(11):4203-13.

160.	Ndzo J, Vuyyuru SK, Trimble T, Yan K, Figueredo G, Moran GW. Association between nutritional status assessed by body mass index and Crohn's disease phenotype: A Nation-wide analysis. *Clinical nutrition ESPEN*. 2024 Dec;64:290-5.

161.	Viganò C, Palermo A, Mulinacci G, Pirola L, Losco A, Meucci G et al. Prevalence of Disease-Related Malnutrition and Micronutrients Deficit in Patients with Inflammatory Bowel Disease: A Multicentric Cross-Sectional Study by the GSMII (Inflammatory Bowel Disease Study Group). *Inflammatory bowel diseases*. 2024 Jul 3;30(7):1112-20.

162.	Viser AC, Cooke AR, Herfarth HH, Anderson C, Proch C, Peery AF. Inflammatory Bowel Disease Patients in the Ambulatory Setting Commonly Screen Positive for Malnutrition. *Gastro hep advances*. 2024;3(2):181-3.

163.	Wei W, Yan P, Zhang Y, Wang Q, Kang J, Liu P et al. Myopenia and body fat distribution in hospitalized ulcerative colitis patients: correlations with clinical characteristics and response to vedolizumab. *Frontiers in nutrition*. 2024;11:1411695.

164.	Xie C, Qian W, Shang L, Wang X, Zhang J, Ma X et al. Assessment of body composition-related imaging parameters indicative of sarcopenia in Chinese patients with Crohn's disease: correlation with disease severity and biologic efficacy. *American journal of translational research*. 2024;16(10):5427-40.

165.	Yu Z, Song W, Ren X, Chen J, Yao Q, Liu H et al. Calcium deficiency is associated with malnutrition risk in patients with inflammatory bowel disease. *Postgraduate medicine*. 2024;136(4):456-67.

166.	Li D, Ye X, Wang M, Huang H, Yan J, Zhang T et al. Clinical characteristics and nutritional status of children with Crohn's disease and risk factors for malnutrition. *Chin J Contemp Pediatr*. 2024;26(11):1194-201(in Chinese).

167.	Ren-juan Liu, Zi-bin Tian, Xue Jing, Ying-jie Guo, Ai-ling Liu, Han-qing Li et al. Association of malnutrition based on Global Leadership Initiative on Malnutrition criteria with the disease activity and adverse clinical outcomes in hospitalized ulcerative colitis patients. *Chin J Clin Nutr*. 2024;32(2).

168.	Yang Liu, Chang-xing Fang, Ying Qiao, Tian L-l. Impact of the third lumbar skeletal muscle index on drug therapy of patients with inflammatory bowel disease. *Chin J Clin Nutr*. 2024;32(2).

169.	Abdoh Q, Al-Amouri F, Kittaneh S, Daoud J, Hamouda S, Badrasawi M. Factors associated with inflammatory bowel disease control among IBD patients in Palestine (2023-2024): A cross-sectional study. *Arab journal of gastroenterology : the official publication of the Pan-Arab Association of Gastroenterology*. 2025 Nov;26(4):425-31.

170.	Hsu WC, Lin SH, Kuo CJ, Chiu HY, Chen CM, Su MY et al. Impact of Obesity on the Long-Term Outcomes of Advanced Therapies in IBD: A Real-World Study in Taiwan. *Journal of inflammation research*. 2025;18:7139-49.

171.	Kim MK, Shin SH, Lee CH, Kim S, Kim JW, Lee S et al. Temporal trends in obesity and its prognostic impact in Korean patients with inflammatory bowel disease. *Journal of gastroenterology*. 2025 May;60(5):583-92.

172.	Kochhar GS, Khataniar H, Hashash JG, Regueiro M, Farraye FA, Desai A. Natural History, Treatment Persistence and Predictors of Treatment Failure in Patients With Ulcerative Colitis on 5-Aminosalicylic Acid: A US Population-Level Cohort Study. *Inflammatory bowel diseases*. 2025 Nov 1;31(11):3069-76.

173.	Liu JF, Jiang QX, Liu J, Liu AL, Wang YH. Investigation and Analysis of Frailty and Nutritional Status in Patients With Inflammatory Bowel Disease. *Crohn's & colitis 360*. 2025 Apr;7(2):otaf010.

174.	Mancone R, Biancone L, Schiavone SC, Fiorillo M, Menna C, Migliozzi S et al. Obesity and Clinical Characteristics of Inflammatory Bowel Disease. *Obesity facts*. 2025;18(5):429-44.

175.	Naruse T, Sato H, Takahashi K, Sato C, Kojima Y, Kawata Y et al. Association between Clinical Characteristics and Sarcopenia or Sarcopenic Obesity in Crohn’s Disease. *Internal Medicine*. 2025;64(10):1451-8.

176.	Oomkens D, Mujagic Z, de Vries A, van der Meulen-de Jong A, Straatmijer T, Löwenberg M et al. Obesity Is Associated with Inferior Clinical Treatment Outcomes in Inflammatory Bowel Disease: A Nationwide Dutch Registry Study. *Digestive diseases and sciences*. 2025 Aug;70(8):2786-93.

177.	Quilliot D, Bonsack O, Mahmutovic M, Peyrin-Biroulet L, Caron B. Exclusion diet and fasting practices in patients with inflammatory bowel disease: Impact on nutritional status. *Clinical nutrition ESPEN*. 2025 Feb;65:375-81.

178.	Rudnicki Y, Calini G, Abdalla S, Colibaseanu D, Larson DW, Mathis KL. Morbid obesity among Crohn's disease patients is on the rise and is associated with a higher rate of surgical complications after ileocolic resection. *Colorectal disease : the official journal of the Association of Coloproctology of Great Britain and Ireland*. 2025 Jan;27(1):e17286.

179.	Shen Y, Liang F, Li Q, Zhang Y, Fu C, Sun X et al. The prevalence and predictors of non-alcoholic fatty liver disease in Chinese patients with inflammatory bowel disease: a multicenter retrospective study. *Scientific reports*. 2025 Dec 28;16(1):515.

180.	Shen Y, Liu H, Liu J, Tang C, Liu Z, Dai X et al. Nutritional status and prevalence of anemia in patients with Crohn's disease after surgery: a multicenter cross-sectional study in China. *Frontiers in nutrition*. 2025;12:1674853.

181.	Youn J, Hsia K, Khadilkar S, Zeina T, Rai P, Rastogi A et al. The Impact of Obesity on the Prevalence and Complications of Perianal Fistulas of Crohn’s Disease. *Digestive diseases and sciences*. 2025;70(1):323-32.

182.	Zhang Q, Wang Y, Liu S, Zhu S, Li P, Zhang S et al. Mortality risk associated with general and central obesity in inflammatory bowel disease patients: a long-term prospective cohort study. *International journal of obesity (2005)*. 2025 Nov;49(11):2303-10.

183.	Wang W, Hu F, Song C, Tian L, Suo Z, Song C et al. Correlation analysis of nutritional status and quality of life in elderly patients with inflammatory bowel disease. *Health Medicine Research and Practice*. 2025;22(9).

184.	Yang Zhongyue, Yajing Z. Correlation between serum NLR, IL-6, TNF-α, and sarcopenia in patients with inflammatory. *International Journal of Digestive Disease*. 2025;45(1).

185.	Bali AS, El Sheikh WG, Ouni A, Kwon J, Moktan V, Chen F et al. Radiographically-Measured Sarcopenia Predicts Hospitalization in Patients With Crohn's Disease. *Journal of clinical gastroenterology*. 2026 Jan 20.

186.	Borsato ROR, Chebli JMF, Ribeiro T, Borsato LHS, Sales MPM, Barra MAL. THE IMPACT OF OBESITY ON THE CLINICAL COURSE OF INFLAMMATORY BOWEL DISEASE. *Arquivos de gastroenterologia*. 2026;63:e25093.

187.	Desalegn Z, Bane A, Merdassa G, Anjajo EA, Minota AA. Prevalence and Determinants of Malnutrition in Ethiopian IBD Patients Using the SGA: A Cross-Sectional Study. *SN Comprehensive Clinical Medicine*. 2026;8(1).

188.	Dharap V, Desai D, Abraham P, Gupta T, Dhoble P, Mehta N et al. Prevalence and outcome of sarcopenia in patients with inflammatory bowel disease: a follow-up study. *Intestinal research*. 2026 Jan;24(1):141-50.

189.	Fang C, Li F, Liu Y, Qiao Y, Tian L. CT-derived sarcopenia and myosteatosis predict treatment escalation in hospitalized patients with inflammatory Bowel disease. *Abdominal radiology (New York)*. 2026 Feb 21.

190.	Fiorindi C, Cei G, Leone S, Previtali E, Burbui G, Celli C et al. Nutritional Risk in Patients with IBD: Results from a National Survey. *Nutrients*. 2026 Apr 16;18(8).

191.	Kaazan P, Seow W, Wilson B, Connor SJ, Andrews JM. The trend of obesity and inflammatory bowel disease in Australia and New Zealand: a bi-nation cohort study. *Internal medicine journal*. 2026 May 5.

192.	Karzan AA, Erdoğan Ç, Akpınar H. Modified Glasgow Prognostic Score as a dual biomarker for disease activity and malnutrition in inflammatory bowel disease. *Scientific reports*. 2026 Apr 29;16(1).

193.	Korwel KM, Lesnovska KP, Herraiz-Adillo Á, Henriksson P, Hjortswang H. Prevalence and risk factors for malnutrition based on the Global Leadership Initiative on Malnutrition (GLIM) criteria in patients with inflammatory bowel disease: a population-based study. *Clinical nutrition ESPEN*. 2026 Jun;73:102988.

194.	Masuda N, Miwa T, Nishimura K, Miwa Y, Kojima K, Onishi S et al. Impact of the global leadership initiative on malnutrition criteria on the quality of life of patients with ulcerative colitis. *Clinical nutrition ESPEN*. 2026 Apr;72:102919.

195.	Nigro E, Marra M, De Santis F, Sammarco R, Leo C, Nardone OM et al. Adiponectin and phase angle in the assessment of sarcopenia in Crohn's disease: beyond muscle mass. *Frontiers in nutrition*. 2026;13:1753532.

196.	Roberts C, Hodges P, Badrulhisham F, Smith R, Clough J, Bewshea C et al. Prevalence and factors associated with obesity in patients with inflammatory bowel disease (IBD) treated with infliximab and vedolizumab across the UK. *Frontline gastroenterology*. 2026.

197.	Xiang Y, Zhou Y, Xu R, Feng Y. Impact of body mass index on length of stay in patients with inflammatory bowel disease: A retrospective study. *Nutrition in clinical practice : official publication of the American Society for Parenteral and Enteral Nutrition*. 2026 Jun 22.

198.	Yu Z, Duan R, Liu Q, Mei W, Chen D, Pan T et al. Comparison of six nutritional screening tools for identifying BMI‑defined malnutrition in patients with Crohn's disease: a cross‑sectional study. *BMC gastroenterology*. 2026 Jul 8.

199.	Zhang Q, Liu H, Liu S, Zhu S, Zhang S, Yang Z et al. Increased Mortality Associated With Sarcopenia in Inflammatory Bowel Disease: A Long-Term Prospective Cohort Study. *The American journal of gastroenterology*. 2026 Jul 1;121(7):1679-89.

200.	Zhong HY, Deng WT, Huang JS, Li JC, Luo XL, Liang CM et al. Evaluating objective nutritional biomarkers for the prediction of sarcopenia in Crohn's disease: development and internal validation of a clinical nomogram. *Frontiers in nutrition*. 2026;13:1838106.

201.	Zhao Z, Ling W, Ma Y, Gong J, Yang Y, Ye X et al. Study on the correlation between phase angle,muscle function,and nutritional status in elective surgery patients with Crohn's disease. *Parenteral & Enteral Nutrition*. 2026;33(1):36-41 (in Chinese).

**Supplementary Table 2. Pooled analysis of malnutrition prevalence under ESPEN, GLIM and SGA/PG-SGA criteria across subgroups**

|  | **ESPEN** | | | | | **GLIM** | | | | | **SGA/PG-SGA** | | | | |
| --- | --- | --- | --- | --- | --- | --- | --- | --- | --- | --- | --- | --- | --- | --- | --- |
|  | **No. Studies** | **Sample size** | **Prevalence (95%CI)** | **I^2^** | **P for subgroup** | **No. Studies** | **Sample size** | **Prevalence (95%CI)** | **I^2^** | **P for subgroup** | **No. Studies** | **Sample size** | **Prevalence (95%CI)** | **I^2^** | **P for subgroup** |
| **Overall** | 10 | 3379 | 24.0 (13.0-40.2) | 100% | / | 20 | 4638 | 36.9 (27.1-47.9) | 98% | - | 10 | 2011 | 25.1 (13.9-41.2) | 98% |  |
| **Sex** |  |  |  |  | 0.84 |  |  |  |  | 0.24 |  |  |  |  | 0.88 |
| Male | 5 | 14031 | 17.85 (5.89-43.00) | 100% |  | 7 | 973 | 31.2 (17.7-48.8) | 95% |  | 2 | 135 | 32.8 (15.8-56.0) | 91% |  |
| Female | 5 | 13296 | 20.88 (6.46-50.23) | 100% |  | 7 | 577 | 45.9 (28.9-63.8) | 93% |  | 2 | 214 | 30.0 (9.0-64.8) | 96% |  |
| **Disease subtype** |  |  |  |  | 0.37 |  |  |  |  | 0.38 |  |  |  |  | 0.51 |
| CD | 7 | 15882 | 25.0 (10.7-48.3) | 100% |  | 9 | 1266 | 34.7 (20.4-52.4) | 97% |  | 8 | 1130 | 29.3 (14.4-50.5) | 97% |  |
| UC | 8 | 12074 | 15.2 (6.9-30.2) | 99% |  | 10 | 1863 | 25.4 (14.9-39.9) | 98% |  | 6 | 462 | 21.4 (10.2-39.4) | 91% |  |
| **Disease Activity** |  |  |  |  | / |  |  |  |  | / |  |  |  |  | / |
| Active | 2 | 366 | 30.05 (25.58-34.95) | 51% |  | / | / | / | / |  | / | / | / | / |  |
| Remission | / | / | / | / |  | / | / | / | / |  | / | / | / | / |  |

BMI body mass index, CD Crohn’s disease, CI confidence interval, ESPEN European Society for Clinical Nutrition and Metabolism, GLIM Global Leadership Initiative on Malnutrition, No. number, PGA-SGA Patient-Generated SGA, SGA Subjective Global Assessment, UC ulcerative colitis, WHO world health organization.

**Supplementary Table 3. Pooled analysis of sarcopenia prevalence under EWGSOP and AWGS criteria across subgroups**

|  | **EWGSOP** | | | | | **AWGS** | | | | |
| --- | --- | --- | --- | --- | --- | --- | --- | --- | --- | --- |
|  | **No. Studies** | **Sample size** | **Prevalence (95%CI)** | **I^2^** | **P for subgroup** | **No. Studies** | **Sample size** | **Prevalence (95%CI)** | **I^2^** | **P for subgroup** |
| **Overall** | 5 | 6151 | 6.2 (2.3-15.5) | 99% | / | 5 | 723 | 29.7 (23.2-37.0) | 80% |  |
| **Sex** |  |  |  |  | 0.62 |  |  |  |  | 0.61 |
| Male | 3 | 377 | 18.98 (6.76-43.09) | 96% |  | 3 | 203 | 30.0 (20.2-41.9) | 77% |  |
| Female | 3 | 292 | 14.00 (6.48-27.67) | 83% |  | 3 | 136 | 35.4 (20.1-54.34) | 83% |  |
| **Disease subtype** |  |  |  |  | 0.54 |  |  |  |  | 0.05 |
| CD | 6 | 2515 | 6.3 (1.9-18.9) | 98% |  | 3 | 348 | 52.9 (16.3-86.7) | 90% |  |
| UC | 4 |  | 3.7 (1.1-11.9) | 96% |  | 3 | 260 | 16.0 (11.0-22.7) | 53% |  |
| **Disease Activity** |  |  |  |  | 0.59 |  |  |  |  | <0.01 |
| Active | 2 | 114 | 19.63 (1.41-80.64) | 97% |  | 3 | 248 | 32.3 (26.7-38.3) | 53% |  |
| Remission | 3 | 655 | 8.99 (1.78-34.95) | 96% |  | 3 | 250 | 17.2 (10.8-26.3) | 59% |  |

AWGS Asian Working Group for Sarcopenia, CD Crohn’s disease, CI confidence interval, EWGSOP European Working Group on Sarcopenia in Older People, No. number, UC ulcerative colitis, WHO world health organization

**Supplementary Table 4. Population-based prevalence of underweight in patients with IBD across subgroups**

|  | **No. Studies** | **Sample size** | **Prevalence (95%CI)** | **I^2^** | **P for subgroup** |
| --- | --- | --- | --- | --- | --- |
| **Overall** | 14 | 108483 | 6.2 (3.4-11.2) | 100% | / |
| **Sex** |  |  |  |  | 0.35 |
| Male | 3 | 6779 | 1.2 (0.5-3.0) | / |  |
| female | 3 | 6956 | 2.2 (1.0-4.8) | / |  |
| **Age** |  |  |  |  | 0.79 |
| Children or adolescent | 3 | 3314 | 8.4 (2.3-26.5) | 99% |  |
| Adult | 9 | 94878 | 6.9 (3.2-14.3) | 100% |  |
| Mixed | / | / | / | / |  |
| **Disease Subtype** |  |  |  |  | 0.50 |
| CD | 9 | 21476 | 10.8 (5.2-21.1) | 100% |  |
| UC | 5 | 5785 | 7.3 (2.9-17.3) | 99% |  |
| **Disease activity** |  |  |  |  | / |
| Active | / | / | / | / |  |
| Remission | / | / | / | / |  |

CD Crohn’s Disease, CI confidence interval, No. number, UC ulcerative colitis, WHO world health organization

**Supplementary Table 5.** **Population-based prevalence of overweight and obesity in patients with IBD across subgroups**

|  | **Overweight** | | | | | **Obesity** | | | | |
| --- | --- | --- | --- | --- | --- | --- | --- | --- | --- | --- |
|  | **No. Studies** | **Sample size** | **Prevalence (95%CI)** | **I^2^** | **P for subgroup** | **No. Studies** | **Sample size** | **Prevalence (95%CI)** | **I^2^** | **P for subgroup** |
| **Overall** | 15 | 131951 | 25.0 (20.5-30.1) | 99% | / | 36 | 1371690 | 14.0 (10.9-17.8) | 100% | / |
| **Sex** |  |  |  |  | 0.05 |  |  |  |  | 0.18 |
| Male | 7 | 13551 | 34.3 (29.5-39.4) | 98% |  | 17 | 375504 | 10.3 (6.7-15.5) |  |  |
| female | 7 | 13288 | 27.5 (23.2-32.3) | 97% |  | 17 | 462976 | 14.9 (10.5-20.9) |  |  |
| **Age** |  |  |  |  | <0.01 |  |  |  |  | <0.01 |
| Children or adolescent | 2 | 6230 | 11.6 (10.8-12.4) | 0% |  | 4 | 19722 | 4.1 (2.0-8.2) | 99% |  |
| Adult | 11 | 115430 | 26.8 (22.7-31.4) | 99% |  | 26 | 1024888 | 15.5 (12.5-19.1) | 100% |  |
| Mixed | / | / | / | / |  | 4 | 316789 | 16.4 (6.2-36.7) | 100% |  |
| **Disease Subtype** |  |  |  |  | 0.23 |  |  |  |  | 0.29 |
| CD | 11 | 35615 | 20.7 (16.0-26.3) | 99% |  | 24 | 398636 | 11.3 (8.3-15.1) | 100% |  |
| UC | 9 | 16818 | 24.6 (21.2-28.4) | 97% |  | 21 | 337435 | 14.2 (10.4-19.0) | 100% |  |
| **Disease activity** |  |  |  |  | / |  |  |  |  | 0.82 |
| Active | / | / | / | / | / | 1 | 2441 | 7.3 (6.3-8.4) | / |  |
| Remission | / | / | / | / | / | 1 | 1563 | 7.5 (6.2-8.2) | / |  |

CD Crohn’s Disease, CI confidence interval, No. number, UC ulcerative colitis, WHO world health organization

**Supplementary Table 6. Prevalence of underweight in patients with IBD at the regional and country level**

|  | **No. Studies** | **Sample size** | **Prevalence (95%CI)** | **I^2^** |
| --- | --- | --- | --- | --- |
| **WHO regions*** |  |  |  |  |
| **Region of the Americas** | 22 | 60613 | 7.6 (5.5-10.5) | 98% |
| USA | 16 | 59129 | 6.6 (4.3-10.0) | 99% |
| Canada | 2 | 554 | 14.8 (12.1-18.0) | 0% |
| Brazil | 4 | 930 | 9.4 (7.6-11.4) | 0% |
| **European Region** | 32 | 48128 | 5.8 (4.2-7.8) | 98% |
| UK | 7 | 38352 | 3.2 (1.6-6.2) | 96% |
| German | 4 | 626 | 7.1 (3.7-13.3) | 83% |
| France | 3 | 2097 | 12.1 (5.9-23.1) | 97% |
| Italy | 6 | 1649 | 9.8 (6.2-15.2) | 93% |
| Netherland | 1 | 417 | 1.4 (0.5-3.1) | / |
| Spain | 2 | 1021 | 2.8 (1.8-4.1) | 59% |
| Switzerland | 1 | 327 | 4.0 (2.1-6.7) | / |
| Turkey | 3 | 1280 | 9.4 (4.8-17.8) | 94% |
| Greece | 1 | 237 | 3.0 (1.2-6.0) | / |
| Hungary | 1 | 1027 | 2.4 (1.6-3.6) | / |
| Israel | 1 | 152 | 18.4 (12.6-25.5) | / |
| Multi-country | 1 | 637 | 10.1 (7.8-12.7) | / |
| **Western Pacific Regions** | 30 | 18535 | 27.0 (20.1-35.2) | 99% |
| Australia | 3 | 7367 | 3.9 (3.4-4.3) | 0% |
| China | 22 | 8197 | 35.4 (28.3-43.3) | 98% |
| Singapore | 1 | 217 | 11.5 (7.6-16.5) | / |
| South Korea | 4 | 2754 | 21.3 (11.8-35.4) | 94% |
| **South East Asia** | / | / | / | / |
| **Eastern Mediterranean Region** | 5 | 1622 | 19.9 (6.0-49.4) | 99% |
| Kuwait | 1 | 528 | 3.4 (2.0-5.3) | / |
| Saudi Arabia | 3 | 978 | 40.6 (16.0-71.1) | 99% |
| Palestine | 1 | 116 | 7.8 (3.6-14.2) | / |
| **African Region** | / | / | / | / |

*Subgroup difference by regions p<0.01

CI confidence interval, IBD inflammatory bowel disease, No. number, WHO World Health Organization

**Supplementary Table 7. Prevalence of consensus-defined malnutrition in patients with IBD at the regional and country level**

|  | **No. Studies** | **Sample size** | **Prevalence (95%CI)** | **I^2^** |
| --- | --- | --- | --- | --- |
| **WHO regions*** |  |  |  |  |
| **Region of the Americas** | 5 | 26281 | 14.5 (5.3-34.0) | 100% |
| USA | 4 | 26036 | 12.9 (3.7-36.2) | 100% |
| Canada | 1 | 245 | 23.3 (18.1-29.1) | / |
| **European Region** | 8 | 2393 | 13.0 (9.4-17.6) | 92% |
| Germany | 1 | 101 | 31.7 (22.8-41.7) | / |
| Greece | 1 | 237 | 11.4 (7.6-16.1) | / |
| Italy | 2 | 453 | 18.3 (12.3-26.3) | 84% |
| Spain | 1 | 33 | 7.5 (4.9-10.9) | / |
| Turkey | 2 | 630 | 9.1 (7.0-11.6) | 0% |
| Sweden | 1 | 306 | 12.4 (8.9-16.6) | / |
| **Western Pacific Regions** | 19 | 4871 | 40.0 (29.5-50.8) | 97% |
| Australia | 1 | 107 | 15.9 (9.5-24.2) | / |
| China | 18 | 4524 | 43.7 (33.4-54.5) | 97% |
| **South East Asia** | 1 | 406 | 25.1 (21.0-29.6) | / |
| India | 1 | 406 | 25.1 (21.0-29.6) | / |
| **Eastern Mediterranean Region** | / | / | / | / |
| **African Region** | 1 | 242 | 51.7 (45.2-58.1) | / |
| Ethiopia | 1 | 242 | 51.7 (45.2-58.1) | / |

*Subgroup difference by regions p<0.01

CI confidence interval, IBD inflammatory bowel disease, No. number, WHO World Health Organization

**Supplementary Table 8. Prevalence of low muscle mass in patients with IBD at the regional and country level**

|  | No. Studies | Sample size | Prevalence (95%CI) | I^2^ |
| --- | --- | --- | --- | --- |
| **WHO regions*** |  |  |  |  |
| **Region of the Americas** | 2 | 280 | 28.3 (22.7-34.7) | 61% |
| USA | 2 | 280 | 28.3 (22.7-34.7) | 61% |
| **European Region** | 5 | 847 | 38.5 (25.3-53.6) | 96% |
| France | 1 | 132 | 40.2 (31.7-49.0) | / |
| Germany | 1 | 114 | 59.7 (50.1-68.7) | / |
| Italy | 2 | 485 | 22.1 (16.0-29.8) | 83% |
| Turkey | 1 | 116 | 53.5 (44.0-62.8) | / |
| **Western Pacific Region** | 18 | 4149 | 53.9 (47.0-60.7) | 91% |
| Australia | 1 | 147 | 36.1 (28.3-44.4) | 83% |
| China | 14 | 2759 | 55.1 (47.5-62.5) | 91% |
| South Korea | 2 | 1139 | 62.5 (52.2-71.7) | 87% |
| Japan | 1 | 104 | 33.7 (24.7-43.6) | / |
| **South East Asia** | 1 | 117 | 40.2 (31.2-49.6) | / |
| India | 1 | 117 | 40.2 (31.2-49.6) | / |
| **Eastern Mediterranean Region** | / | / | / | / |
| **African Region** | / | / | / | / |

*Subgroup difference by regions p<0.01

CI confidence interval, IBD inflammatory bowel disease, No. number, WHO World Health Organization

**Supplementary Table 9. Prevalence of sarcopenia in patients with IBD at the regional and country level**

|  | **No. Studies** | **Sample size** | **Prevalence (95%CI)** | **I^2^** |
| --- | --- | --- | --- | --- |
| **WHO regions*** |  |  |  |  |
| **Region of the Americas** | / | / | / |  |
| **European Region** | 3 | 710 | 4.9 (2.2-10.5) | 85% |
| Greece | 1 | 237 | 2.1 (0.7-4.9) | / |
| Turkey | 2 | 473 | 7.9 (4.6-13.3) | 71% |
| **Western Pacific Regions** | 6 | 934 | 28.9 (20.9-38.5) | 88% |
| Australia | 1 | 137 | 12.4 (7.4-19.1) | / |
| China | 5 | 797 | 33.0 (26.7-40.0) | 80% |
| **South East Asia** | 1 | 114 | 21.9 (14.7-30.7) | / |
| India | 1 | 114 | 21.9 (14.7-30.7) | / |
| **Eastern Mediterranean Region** | / | / | / | / |
| **African Region** | / | / | / | / |

*Subgroup difference by regions p<0.01

CI confidence interval, IBD inflammatory bowel disease, No. number, WHO World Health Organization

**Supplementary Table 10. The odds ratios (ORs) for the prevalence of nutritional disorders in patients with inflammatory bowel disease grouped by WHO regions**

|  | **Underweight** | | **Malnutrition** | | **Overweight** | | **Obesity** | | **Low muscle mass** | | **Sarcopenia** | |
| --- | --- | --- | --- | --- | --- | --- | --- | --- | --- | --- | --- | --- |
|  | **OR (95%CI)** | **P** | **OR (95%CI)** | **P** | **OR (95%CI)** | **P** | **OR (95%CI)** | **P** | **OR (95%CI)** | **P** | **OR (95%CI)** | **P** |
| **WHO regions** |  |  |  |  |  |  |  |  |  |  |  |  |
| Region of the Americas | Ref |  | Ref |  | Ref |  | Ref |  | 0.64 (0.24-1.69) | 0.36 | / | / |
| European Region | 0.74 (0.43-1.29) | 0.02 | 0.87 (0.30-2.49) | 0.79 | 0.89 (0.65-1.23) | 0.49 | 0.66 (0.46-0.94) | 0.02 | Ref |  | Ref |  |
| Western Pacific Regions | 4.52 (2.60-7.87) | <0.01 | 3.90 (1.55-9.83) | <0.01 | 0.49 (0.32-0.76) | <0.01 | 0.48 (0.31-0.76) | <0.01 | 1.87 (1.04-3.37) | 0.04 | 11.30 (4.41-29.00) | <0.01 |
| South East Asia | / | / | 1.98 (0.26-14.81) | 0.51 | / | / | / | / | 1.07 (0.30-3.86) | 0.91 | 7.78 (1.44-42.07) | 0.02 |
| Eastern Mediterranean Region | 3.09 (1.16-8.22) | 0.02 | / | / | 0.89 (0.48-1.68) | 0.73 | 0.74 (0.29-1.93) | 0.54 | / | / | / | / |
| African Region | / | / | / | / | / |  | / | / | / | / | / | / |

CI confidence interval, NA non-available, OR odds ratio, Ref reference, WHO world health organization

**Supplementary Table 11. Prevalence of overweight in patients with IBD at the reginal and country level**

|  | **No. Studies** | **Sample size** | **Prevalence (95%CI)** | **I^2^** |
| --- | --- | --- | --- | --- |
| **WHO regions*** |  |  |  |  |
| **Region of the Americas** | 25 | 86379 | 26.2 (22.8-30.0) | 98% |
| Brazil | 4 | 968 | 31.7 (26.3-37.6) | 74% |
| USA | 21 | 85411 | 25.2 (21.5-29.3) | 98% |
| **European Region** | 29 | 47853 | 24.0 (19.4-29.1) | 97% |
| Denmark | 1 | 210 | 24.3 (18.7-30.7) | / |
| France | 2 | 1844 | 20.5 (14.6-28.2) | 83% |
| Germany | 2 | 411 | 32.4 (24.9-40.9) | 84% |
| Greece | 1 | 237 | 34.6 (28.6-41.0) | / |
| Italy | 5 | 964 | 21.1 (16.2-27.3) | 81% |
| Netherland | 2 | 1483 | 31.3 (26.8-36.2) | 85% |
| Poland | 1 | 675 | 3.6 (2.3-5.2) | / |
| Spain | 2 | 1021 | 31.9 (27.6-36.5) | 76% |
| Sweden | 1 | 306 | 33.4 (28.7-39.6) | / |
| Switzerland | 1 | 327 | 6.7 (4.3-10.0) | / |
| Turkey | 3 | 1280 | 15.2 (4.2-42.6) | 99% |
| UK | 6 | 38458 | 33.4 (28.9-38.1) | 98% |
| Multi-countries | 1 | 637 | 17.9 (15.0-21.1) | / |
| **Western Pacific Regions** | 11 | 14517 | 14.9 (11.0-19.9) | 98% |
| Australia | 2 | 7263 | 32.1 (31.1-33.2) | 61% |
| China | 6 | 4605 | 11.5 (8.0-16.2) | 92% |
| South Korea | 3 | 2649 | 15.8 (12.1-20.5) | 78% |
| **South East Asia** | / | / | / | / |
| **Eastern Mediterranean Region** | 4 | 1453 | 24.1 (19.3-29.7) | 84% |
| Kuwait | 1 | 528 | 28.2 (24.4-32.3) | / |
| Saudi Arabia | 2 | 809 | 21.3 (14.8-29.6) | / |
| Palestine | 1 | 116 | 26.7 (18.9-35.7) | / |
| **African Region** | / | / | / | / |

*Subgroup difference by regions p<0.01

CI confidence interval, IBD inflammatory bowel disease, No. number, WHO World Health Organization

**Supplementary Table 12. Prevalence of obesity in patients with IBD at the reginal and country level**

|  | **No. Studies** | **Sample size** | **Prevalence (95%CI)** | **I^2^** |
| --- | --- | --- | --- | --- |
| **WHO regions*** |  |  |  |  |
| **Region of the Americas** | 37 | 1240130 | 16.5 (13.8-19.6) | 100% |
| Brazil | 4 | 968 | 16.2 (13.3-19.5) | 55% |
| Canada | 3 | 7572 | 11.2 (6.9-17.7) | 90% |
| USA | 30 | 1231590 | 17.2 (14.0-21.0) | 100% |
| **European Region** | 27 | 74213 | 11.0 (8.3-14.6) | 99% |
| Denmark | 1 | 210 | 21.4 (16.1-27.6) | / |
| France | 3 | 3909 | 8.5 (3.6-18.7) | 98% |
| Germany | 2 | 411 | 18.6 (11.8-28.0) | 88% |
| Greece | 1 | 237 | 27.0 (21.5-33.1) | / |
| Hungary | 1 | 1027 | 3.3 (2.3-4.6) | / |
| Israel | 1 | 25269 | 23.0 (22.4-23.5) | / |
| Italy | 4 | 664 | 10.3 (5.6-18.1) | 88% |
| Netherland | 1 | 417 | 11.5 (8.6-15.0) | / |
| Poland | 1 | 675 | 4.9 (3.4-6.8) | / |
| Spain | 2 | 1021 | 17.0 (14.9-19.5) | 0% |
| Switzerland | 2 | 2377 | 10.3 (5.2-19.5) | 95% |
| Turkey | 2 | 456 | 11.4 (8.8-14.7) | 39% |
| UK | 5 | 36903 | 10.8 (4.3-24.8) | 100% |
| Multi-countries | 1 | 637 | 4.1 (2.7-5.9) | / |
| **Western Pacific Regions** | 12 | 19061 | 7.3 (3.1-16.3) | 99% |
| Australia | 3 | 980 | 25.1 (16.5-36.3) | 93% |
| China | 3 | 1097 | 1.2 (0.4-3.6) | 69% |
| Japan | 1 | 183 | 15.9 (10.9-22.0) | / |
| South Korea | 5 | 16801 | 8.3 (2.9-21.9) | 100% |
| **South East Asia** | / | / | / | / |
| **Eastern Mediterranean Region** | 2 | 963 | 14.6 (11.7-18.0) | 74% |
| Kuwait | 1 | 528 | 12.5 (9.8-15.6) | / |
| Saudi Arabia | 1 | 435 | 17.0 (13.6-20.9) | / |
| **African Region** | / | / | / | / |

*Subgroup difference by regions p=0.04

CI confidence interval, IBD inflammatory bowel disease, NA none available, No. number, WHO World Health Organization

**Supplementary Figures**

**Supplementary Figure 1. Publication bias assessment of the pooled estimates of underweight in patients with inflammatory bowel disease. A**. Assessment of publication bias using funnel plot. **B**. Funnel plot after tri-and-fill adjustment

**Supplementary Figure 2. Publication bias assessment of the pooled estimates of consensus-defined malnutrition in patients with inflammatory bowel disease.**

**Supplementary Figure 3. Publication bias assessment of the pooled estimates of overweight and obesity in patients with inflammatory bowel disease. A**. Funnel plot of pooled estimated of overweight. **B**. Funnel plot of pooled estimated of overweight after tri-and-fill adjustment; **C**. Funnel plot of pooled estimated of obesity.

**Supplementary Figure 4. Forest plot of the prevalence of visceral obesity in patients with inflammatory bowel disease.** CI confidence interval, GLMM generalized linear mixed model

**Supplementary Figure 5. Funnel plot of pooled prevalence of low muscle mass in patients with inflammatory bowel disease.**

**Supplementary Figure 6. Forest plot of the pooled prevalence rate of low muscle mass with obesity in patients with inflammatory bowel disease.** CI confidence interval, GLMM generalized linear mixed model

**Supplementary Figure 7. Pooled prevalence rate of malnutrition grouped by consensus criteria (GLIM, ESPEN, MNA and SGA/PG-SGA).** CI confidence interval, ESPEN European Society for Clinical Nutrition and Metabolism, GLIM Global Leadership Initiative on Malnutrition, GLMM generalized linear mixed model, MNA Mini Nutritional Assessment, SGA Subjective Global Assessment, PG-SGA Patient-Generated- Subjective Global Assessment

**Supplementary Figure 8. Forest plot of the prevalence of malnutrition based on ICD coding**. CI confidence interval, GLMM generalized linear mixed model

**Supplementary Figure 9. Forest plot of the pooled prevalence of sarcopenia grouped by specific criteria**. AWGS Asian Working Group of Sarcopenia, CI confidence interval, EWGSOP European Working Group of Sarcopenia in Older Patients, GLMM generalized linear mixed model
